# The fossil teeth of the Peking Man

**DOI:** 10.1038/s41598-018-20432-y

**Published:** 2018-02-01

**Authors:** Song Xing, María Martinón-Torres, José María Bermúdez de Castro

**Affiliations:** 1 Key Laboratory of Vertebrate Evolution and Human Origins of Chinese Academy of Sciences, Institute of Vertebrate Paleontology and Paleoanthropology, Chinese Academy of Sciences, Beijing, 100044 China; 2 National Research Center on Human Evolution (CENIEH), Paseo de la Sierra de Atapuerca 3, 09002 Burgos, Spain; 3 University College London (UCL) Anthropology, 14 Taviton Street, London, WC1H 0BW UK

## Abstract

This study provides new original data, including the endostructure of most Zhoukoudian *H. erectus* teeth preserved to date, since the publication of Black in 1927 and Weidenreich in 1937. The new evidence ratifies the similarities of Zhoukoudian with other East Asian mid-Middle Pleistocene hominins such as Hexian and Yiyuan, and allows defining a dental pattern potentially characteristic of this population commonly referred to as classic *H. erectus*. Given the possible chronological overlaps of classic *H. erectus* with other archaic *Homo*, the characterization of this group becomes a key issue when deciphering the taxonomy and evolutionary scenario of the Middle Pleistocene hominins in East Asia. Internally, the most remarkable feature of Zhoukoudian teeth is the highly crenulated enamel-dentine junction (EDJ) and its imprint on the roof of the pulp cavity. So far, this “dendrite-like” EDJ has been found only in East Asia Middle Pleistocene hominins although a large group of samples were assessed, and it could be useful to dentally define classic *H. erectus* in China. The crenulated EDJ surface, together with the stout roots and the taurodontism could be a mechanism to withstand high biomechanical demand despite a general dentognathic reduction, particularly of the crowns, in these populations.

## Introduction

The “Peking Man” from Zhoukoudian Locality 1, Beijing, China, is one of the earliest and most emblematic hominins ever found in human history^1^. It refers to *Sinanthropus pekinensis* (now usually lumped into the *Homo erectus* taxon) named by Black in 1927^1^. The evolutionary interpretation of the Zhoukoudian hominin materials has changed throughout the years. The primary description and comparison of the Zhoukoudian materials^1–5^ filled, at that time a perceived gap between ape and man, and they were crucial for the characterization of the species *H. erectus*^6–8^. Later studies suggested that Zhoukoudian had a less central role in our evolutionary story, representing no more than a side branch in our “family tree”^9–11^. Some researchers have even argued that Zhoukoudian sample may not be a good representative of the *H. erectus* taxon given the distinctive cranial differences they have when compared to the Java counterparts the species was named on^12–14^.

The problem is that, for many decades, the name *H. erectus* has been used as a blanket term to refer to almost any hominin found in Asia during the Pleistocene until the appearance of *Homo sapiens*. Recent fossil and genetic data suggested that the taxonomies of the Asian hominins may have been oversimplified. In particular, discoveries like the Xuchang crania in North China^15^ or the reassessment of fossils samples such as Xujiayao^16–18^, Maba^19^ or Panxian Dadong^20^ reinforce the idea that other hominin lineages different from *H. erectus* may have lived in continental Asia during the same period. These fossils present some primitive features in common with other *H. erectus* samples: the Maba endocast is narrow at the frontal lobes and short and flattened in the parietal areas^19^; the Xuchang neurocrania is low and inferiorly broad^15^; the Xujiayao sample comprises a thick and strongly built cranium, and large and complex molars^16,21^ and the Panxian Dadong upper central incisor displays conspicuous finger-like projections at its lingual surface^20^. While displaying a group of ancestral features, these hominins also show derived features that approximate them to *H. sapiens*. In this aspect, it is noteworthy the neurocranial enlargement and gracilization in the Xuchang specimen^15^; a high and rounded temporal squama, simplified occlusal and smooth buccal surfaces of upper premolars, and a symmetrical crown outline with a pronouncedly reduced lingual cusp of P^3^ in the Xujiayao sample^16,21^; derived positions of the frontal lobes in relation to the orbits and morphologies of frontal sinus and the frontal squama in Maba^19^; and modern human-like crown outline shape of upper and lower premolars and incisor-like lower canine in Panxian Dadong^20^. In addition, Xuchang and Xujiayao have some features that were classically found in the Neanderthal lineage. Xuchang displays a suprainiac fovea and its nuchal torus and temporal labyrinth have been described as Neanderthal-like^15^. Similarly, the morphologies of Xujiayao’s temporal labyrinth and mandible also resemble those of Neanderthals^22^. In addition, fossils like Dali and Yunxian have been referred to as “archaic” or “post-erectus” hominins and possible representatives of *H. heidelbergensis* taxon^23–25^. These, together with the Xujiayao or the Xuchang hypodigm, could be potential candidates to represent the phenotypically “elusive” Denisovans^16,26^. Given a taxonomically more diverse context for the Middle Pleistocene in Asia, the identification and definition of morphological features that can define *H. erectus* in China, become an issue of central importance to understand the evolutionary story of the genus *Homo* in continental Asia.

Unfortunately, the majority of the Zhoukoudian fossils unearthed before 1937 were lost during World War II. As a consequence, most of the studies and discussions about this paramount sample, in the last 80 years, have been solely based on casts and on the descriptions and drawings made by of Weidenreich in 1930 s and 1940 s^2–5^. This has prevented the applications of the latest technologies developed in the field of virtual anthropology, such as microtomography (micro-CT).

After World War II, three systematic excavations were developed in Zhoukoudian Locality 1^27–29^. The excavations from 1949-1959 provided five isolated teeth and one mandible^27,28^. Another isolated tooth was found in 1966^29^. These six teeth provide us the opportunity to restudy and characterize the dental features of the Zhoukoudian using original fossils, instead of casts and descriptions. Here, we provide new original data, including a detailed and comprehensive study of the endostructure of most Zhoukoudian teeth preserved to date through the application of microcomputed tomography (micro-CT). The teeth are compared against a large *Homo* sample from Europe, Asia and Africa including modern humans and some unpublished Middle Pleistocene fossils from Asia. The original Zhoukoudian sample presented here consists of 6 original fossil teeth, including I^1^ (PA66), P^3^ (PA67), P^4^ (PA68), P_3_ (PA110), M_1_ (PA69), and M_2_ (PA70). Our comparison will has a special focus on other *H. erectus sensu lato* (*s.l*.) from China, Java, Dmanisi and Africa. *H. erectus s.l*. is used here to refer to the Early and Middle Pleistocene *Homo* specimens of Africa/West Asia (also called *H. ergaster, Telanthropus capensis*, *Homo leakeyi*, *Atlanthropus mauritanicus*) and East Asia (often called “classic *H. erectus*”). In order to assess the Zhoukoudian’s affinities with other hominins, we performed morphological comparisons of both external (outer enamel surface or OES) and internal (enamel-dentine junction or EDJ and pulp cavity) features, as well as geometric morphometric analysis of the crown outline shape.

## Results

Here we will summarize the morphologies of the 6 Zhoukoudian fossil teeth and their comparisons with other *H. erectus s.l*. A detailed description and comparison of each tooth can be found in the Description of dental morphologies and Comparative dental morphology of the SI Text and SI Table 1 of the Supplementary Information.

**Figure 1:**
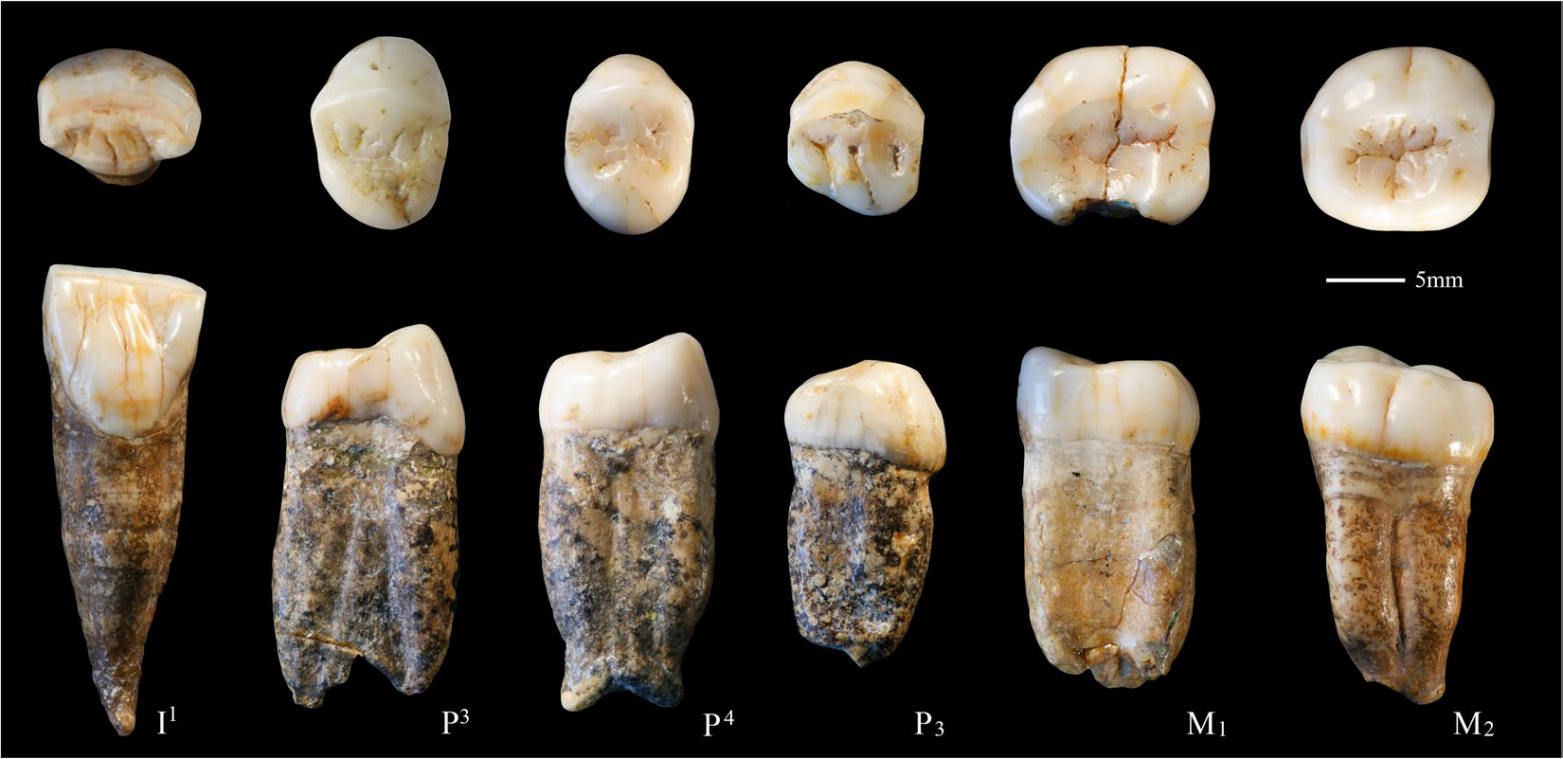
The fossil teeth from Zhoukoudian Locality 1. From left to right are PA66, PA67, PA68, PA110, PA69, and PA70.

**Figure 2:**
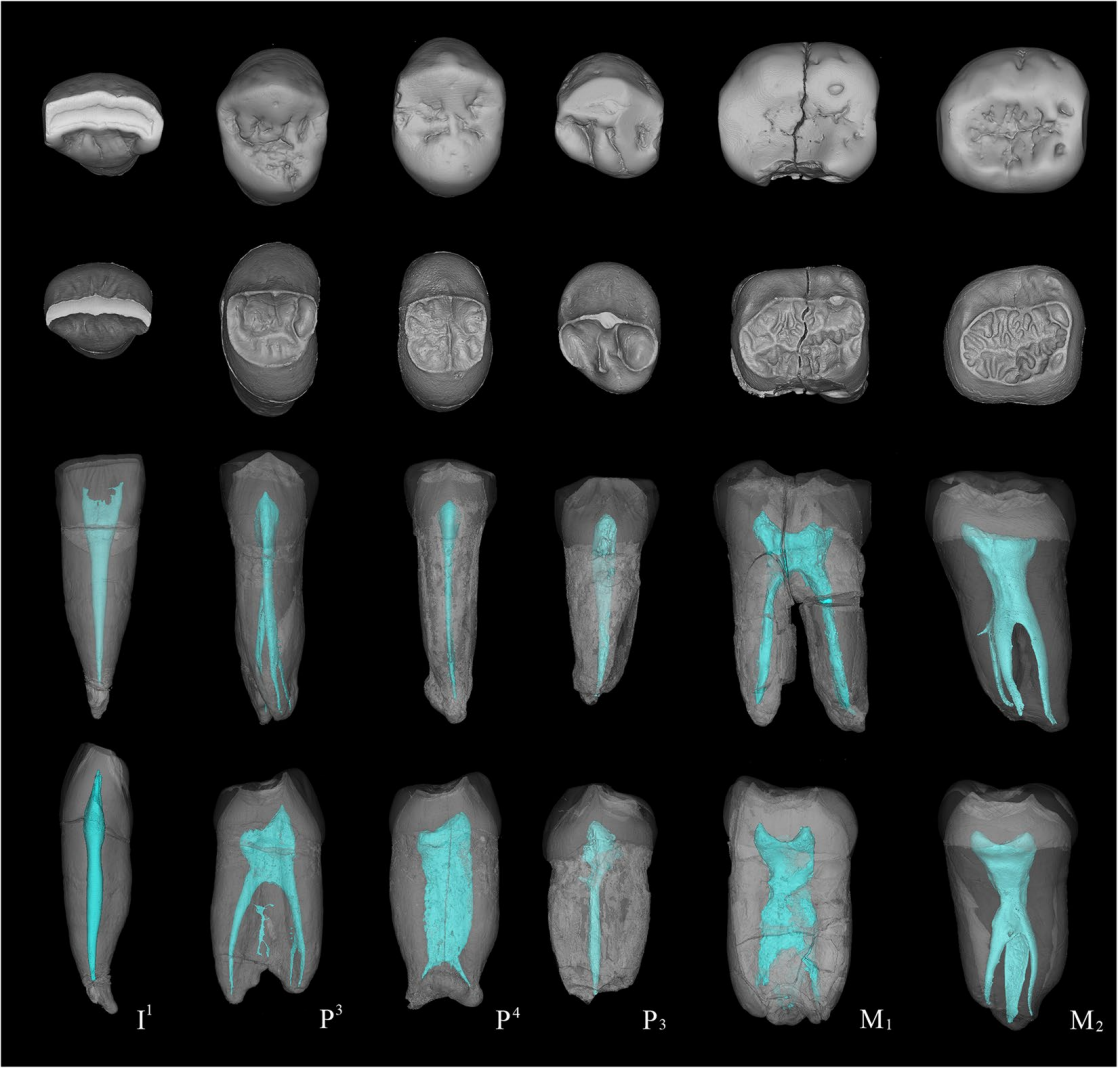
3D virtual reconstructions of the Zhoukoudian fossil teeth. First row: occlusal view of the enamel surface; second row: occlusal view of the dentine surface; third row: buccal view of the whole tooth with the enamel and dentine being transparent and pulp cavity being opaque; forth row: mesial view of the whole tooth with the enamel and dentine being transparent and pulp cavity being opaque. From left to right are PA66, PA67, PA68, PA110, PA69, and PA70.

**Figure 3:**
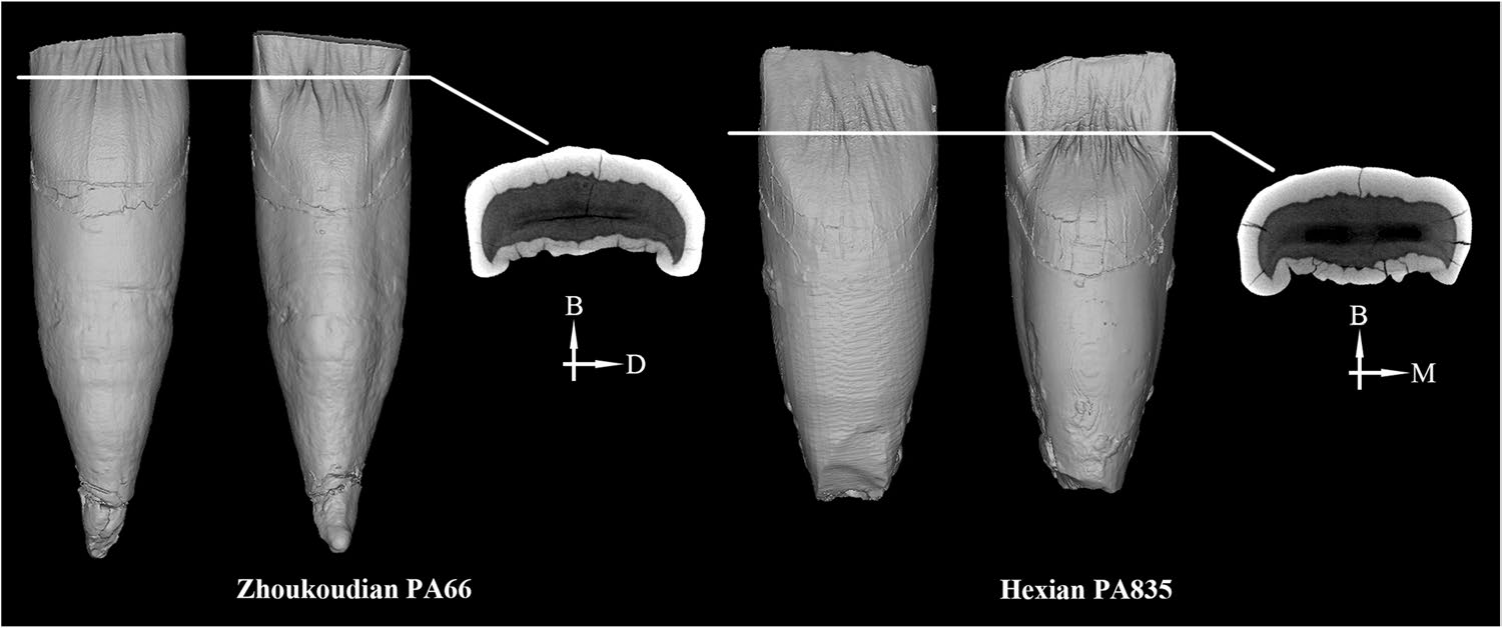
Comparisons of Zhoukoudian and Hexian I^1^s in the features of dentine surface. B: buccal; D: distal; M: mesial. Note the crenulations of the dentine at the labial surface.

**Figure 4:**
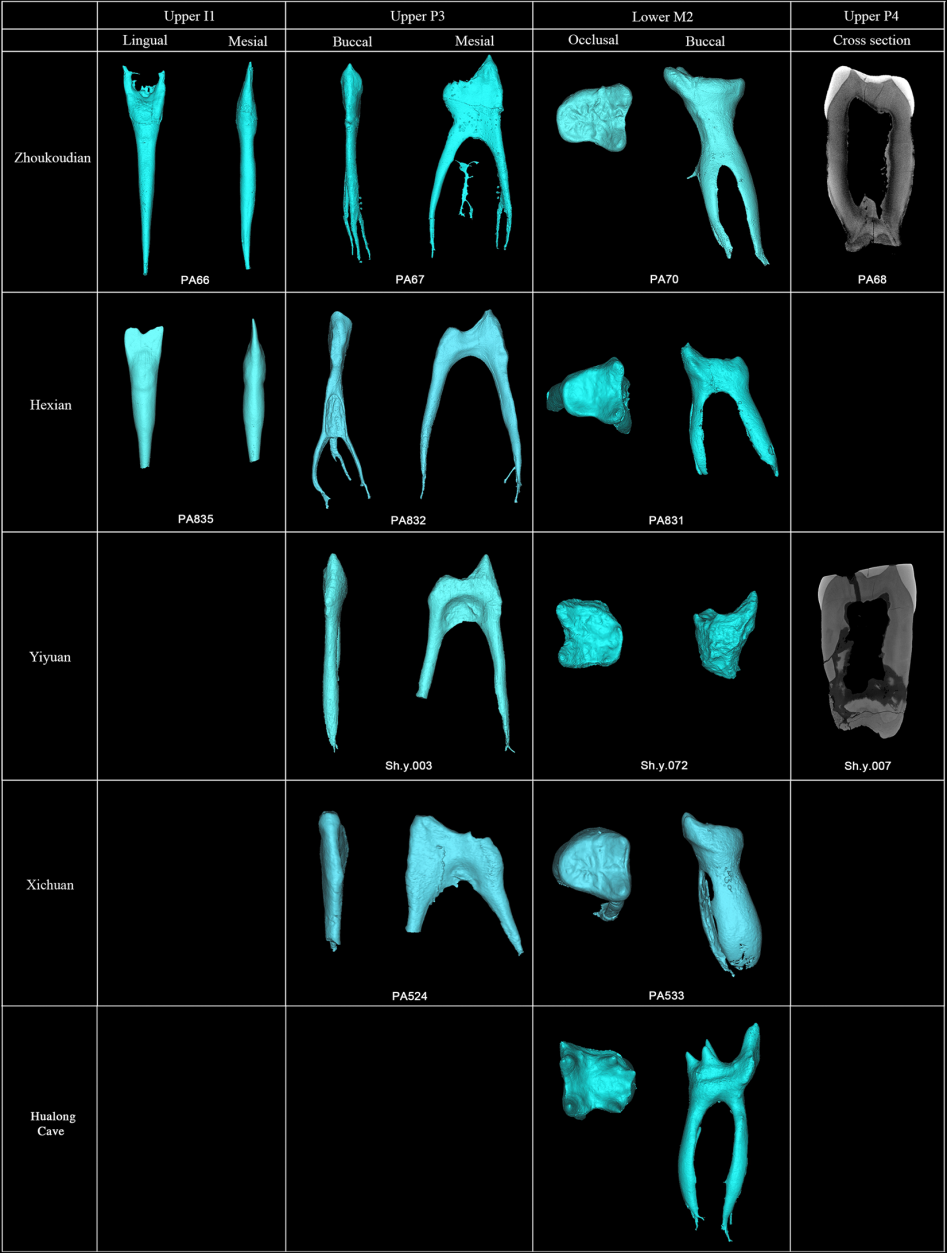
Comparisons of Zhoukoudian, Hexian, Yiyuan, Xichuan, Hualong Cave I^1^s, P^3^, P^4^, and M_2_s in the morphologies of pulp cavity.

PA66 (Left I^1^) (Fig. 1), as the other Zhoukoudian I^1^s^4^ (See also SI Figs 1 and 2), displays a combination of features, including pronounced shoveling, a moderately convex labial surface, a strong basal eminence, and several lingual finger-like prolongations. The pronounced shoveling found in Zhoukoudian could also be identified in Hexian^30^, and is more developed than in African *H. ergaster*, Dmanisi, and Sangiran hominins^31–33^. The finger-like prolongations are absent from the Sangiran specimens, but appear in African *H. ergaster*, such as KNM-WT 15000^31,32^. One of the most remarkable features of PA66 is that the expression of buccal wrinkles and lingual ridges at the OES is also present at the EDJ and the surface of pulp cavity (Figs. 2, 3 and 4). The same pattern can also be detected in the Hexian I^1^ (Figs 3, 4).

PA67 (Right P^3^) is characterized by an asymmetrical crown outline, a continuous transverse crest, and bifurcated essential crest of the buccal cusp (Fig. 1 and SI Fig. 1). The occurrence of a transverse crest, as in PA67 is more frequent in Asian than in African *H. erectus s.l*. and can also be found in ZKD16, Dmanisi D3672, Sangiran 4, Sangiran 7–35, Xichuan PA543, and Hexian PA832^4,30–33^. Although PA67 has externally coalesced buccal and lingual roots with bifid apices, the 3D reconstruction reveals three independent root canals so it should be classified as three-rooted type (Figs 2 and 4). Most of *H. erectus s.l*. present two-rooted P^3^ (coalesced or not)^31–33^ (SI Fig. 3). Three-rooted P^3^s like that of Zhoukoudian are only found in a few cases like Hexian PA832, Sangiran 7–35 and African *H. ergaster* KNM-ER 1808^30–32^.

The crown contour of PA68 (Right P^4^) is ellipse-like and slightly asymmetrical, and the widths of the buccal and lingual cusps are roughly equal (Fig. 1 and SI Fig. 1). This type of crown outline shape is typical of other *H. erectus s.l*. specimens (SI Fig. 4)^4,31,32^. The presence of a continuous transverse crest, like that in PA68, also exists in other specimens of *H. erectus s.l*., such as ZKD 27, Sangiran 7–29, and KNM-ER 3733^4,31,32^. The root is wide and comprises two radicals that coalesce along most of its length except for a strongly bifurcated tip. The number of premolar roots in *H. erectus s.l*. ranges from two to three roots, and in some cases the buccal and lingual roots are coalesced as in PA68^31–33^. An enlarged pulp cavity (hypertaurodont; term from Shaw [1928]^34^), as shown in the Zhoukoudian PA68, can also be observed in the East Asian Middle Pleistocene hominin from Yiyuan (Sh.y. 007) (Fig. 4).

The crown contour of PA110 (Right P_3_) is asymmetrical with a slightly protruding distolingual corner (Fig. 1 and SI Fig. 5). As shown in SI Fig. 6, Zhoukoudian specimens cluster with most specimens of Sangiran, Tighenif, Atapuerca TD6, KNM-WT 15000, and KNM-ER 992. D211 and D2375 from Dmanisi display a more mesiodistally elongated and asymmetrical crown outline than the Zhoukoudian P_3_s, and they cluster with early *Homo* specimens. Compared to Zhoukoudian, the crown outline of Sangiran 9 is more buccolingually elongated. Although incomplete, the root is single, with a single canal, and weakly-developed mesial and distal longitudinal grooves. Like PA110, there are other single-rooted specimens in the Zhoukoudian sample (ZKD21 and ZKD23)^4^ (See also SI Fig. 7). ZKD82 and ZKD85 display a Tomes’ root with two mesial and one distal longitudinal groove like Trinil 5, Lantian mandible, and D211^33^. Apart from the single-rooted Tomes’ roots, more complex root structures, like two-rooted premolars can be observed in other members of *H. erectus s.l*. from Dmanisi (D2600), East Africa (KNM ER-730), and Sangiran (Sangiran 5, 8, and 9)^31,33,35^.

The Zhoukoudian M_1_s, as most specimens from Sangiran and Tighenif, tend to have a relatively wider crown contour than *H. ergaster* (SI Fig. 8, SI Table 2). The boxplots of the crown indices (BL*100/MD) show that the median value of Zhoukoudian *H. erectus* is higher than that of African early *Homo*, *H. ergaster*, and Dmanisi, although their ranges of variation overlap (SI Fig. 9). An independent t-test reveals a significant difference in the crown index between Zhoukoudian and both African early *Homo* and *H. ergaster* (p < 0.05 in each case) (SI Table 2). Besides, the Zhoukoudian M_1_s are characterized by having a crown outline that is wider in the mesial aspect (more buccolingually elongated trigonid than the talonid) (SI Fig. 8). This crown contour could also be seen in Lantian and Sangiran 22^35^, but is rarely seen in other specimens of *H. erectus s.l*.

Unlike the Neanderthal-lineage^36,37^, trigonid crests are uncommon in the Zhoukoudian M_1_s sample, except for continuous mesial trigonid crest (MeTC) in ZKD34 (SI Fig. 8). A middle trigonid crest can be identified in Xichuan PA531, some Sangiran (e.g., Sangiran 6, Sangiran 7–43) and Dmanisi specimens (D211 and D2735), but not in African *H. ergaster*^31–33,35^.

**Figure 5:**
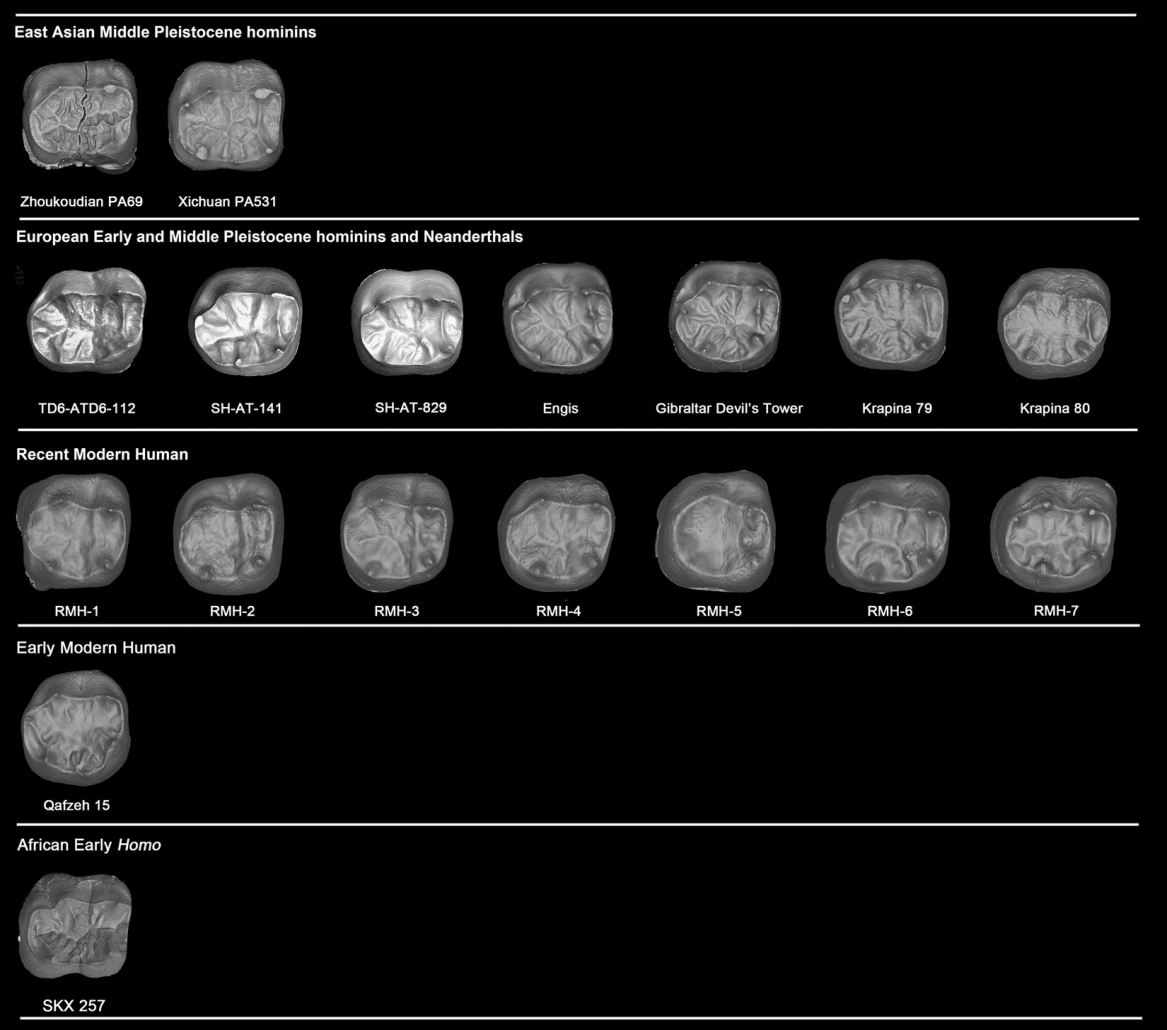
Comparison of M_1_ dentine surfaces of Zhoukoudian and other comparative specimens. TD6 level of Atapuerca-Gran Dolina; SH: Atapuerca-Sima de los Huesos; RMH: Recent modern human. Some of the dental images were mirror-imaged to facilitate the comparison with the Zhoukoudian M_1_. CT data of Engis, Gibraltar, and Qafzeh are from European Synchrotron Radiation Facility (ESRF) (http://paleo.esrf.fr) and published in Smith *et al*.^67^. Data of Krapina is from NESPOS^68^. Picture of SKX 257 was revised after Pan^69^.

The EDJ surface of Zhoukoudian PA69 is highly crenulated mainly due to the development of several secondary ridges that accompany the essential crest of the cusps and that has been defined as “dendrite-like” (Fig. 2). This pattern is similar to that of Hexian and Yiyuan molars. The EDJ of *Au. africanus* (n = 1), *P. robustus* (n = 4), early *Homo* (n = 1) is simpler and relatively smoother (Lei Pan pers. comm. and Fig. 5). Forty-two M_1_s of European Early and Middle Pleistocene hominins and Neanderthals display simpler EDJ surface than those of *H. erectus* in China, although the EDJ surface of Engis and Gibraltar M_1_s are relatively more complex and exhibit more accessory ridges than the rest of the *H. neanderthalensis* sample. In both fossil and recent *H. sapiens* (n = 28), we did not observe the “dendrite-like” EDJ surface (Fig. 5 and SI Fig. 10). The EDJ pattern is also more complex than that present in earlier hominins (*Australopithecus* and *Paranthropus*) and genus *Homo* available in the literature^38–41^.

On the EDJ surface of PA69, a mesial protoconid ridge is present in the mesial aspect of the protostylid and forms a protostylid-protoconid shelf combination (Fig. 2). This structure is also present in Xichuan PA531, but less obvious on the Tighenif 2^41^ and absent in MA93 from East African late Early Pleistocene^42^. From a lateral view, the width of the roots in PA69 and other Zhoukoudian M_1_s does not decrease clearly until the very apical end (Fig. 1 and SI Fig. 8), a trait that has been described as typical of classic *H. erectus* from China and Java^30,43^.

The Zhoukoudian M_2_s are characterized by a round crown outline where both the trigonid and the talonid are buccolingually expanded relative to its length. In addition, there is no constriction along the crown contour to separate trigonid and talonid (See also SI Fig. 11). The median value of range of variation for the crown index (BL*100/MD) of Zhoukoudian M_2_s is higher than all samples involved in the present study except for the East Asian Middle Pleistocene hominins (Hexian and Yiyuan) and European early modern humans (SI Fig. 12). In addition, Zhoukoudian’s median value exceeds the upper limit of variation of East Asian Early Pleistocene (Sangiran Bapang-AG), *H. ergaster*, and African early *Homo* (SI Fig. 12). The independent t-test shows that the differences in the crown index between Zhoukoudian and *Australopithecus* (p < 0.01), African early *Homo* (p < 0.05), European Middle Pleistocene hominins (p < 0.05), and recent modern humans (p < 0.01) are significant (SI Table 3). Geometric morphometric analysis further confirms the peculiarity of the crown outline shape of Zhoukoudian M_2_s. As seen in SI Fig. 13, Zhoukoudian sample only shares a small area along the upper border of the Neanderthals’ distribution area, and does not overlap with European Early and Middle Pleistocene hominins. The Sangiran specimens fall between *H. ergaster* and East Asia Middle Pleistocene *H. erectus* and overlap with both of them.

A continuous MTC, typical in Neanderthal-linear specimens, can be found in isolated specimens from African (KNM-ER 1808), Dmanisi (D2735), and Sangiran (Sangiran 9)^31,33,35^. However, this feature is absent in the Zhoukoudian M_2_s, except for a continuous MeTC in PA70 (Figs 1 and 2, SI Fig. 11).

**Figure 6:**
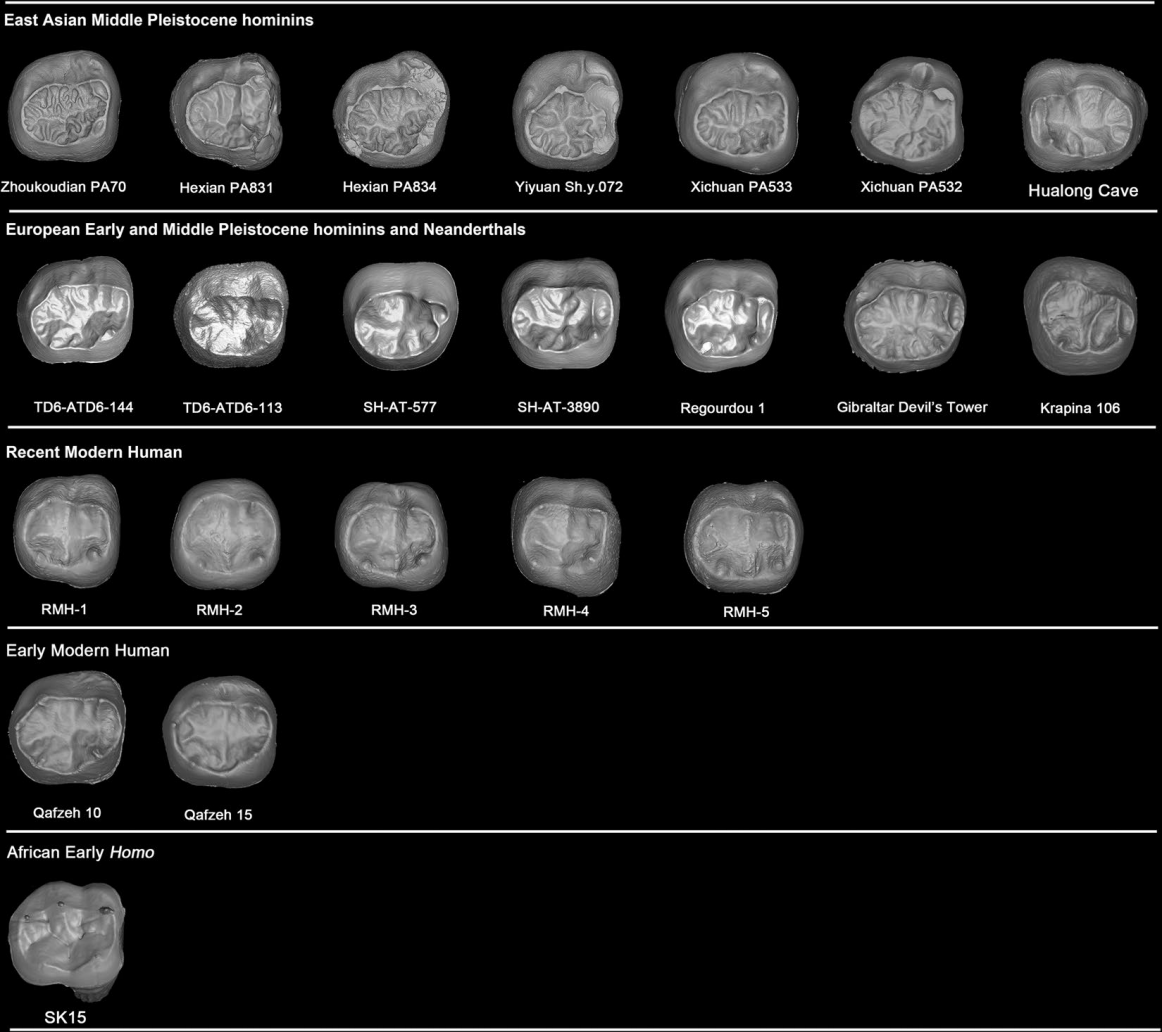
Comparison of M_2_ dentine surfaces of Zhoukoudian and other comparative specimens. TD6 level of Atapuerca-Gran Dolina; SH: Atapuerca-Sima de los Huesos. RMH: Recent modern human. Some of the dental images were mirror-imaged to facilitate the comparison with the Zhoukoudian M_2_. CT data of Engis, Gibraltar, and Qafzeh are from European Synchrotron Radiation Facility (ESRF) (http://paleo.esrf.fr) and published in Smith *et al*.^67^. Data of Regourdou and Krapina is from NESPOS^68^. Picture of SK15 was revised after Pan^69^.

The most remarkable feature of the Zhoukoudian PA70 is its “dendrite-like” EDJ surface, highly crenulated, with interconnected ridges, bifurcated essential crests, accessory ridges, and accessory cusps. The degree of EDJ complexity in Zhoukoudian PA70, similar to Hexian PA839 and Yiyuan Sh.y.072^30,44^, is more pronounced than in PA69 (Zhoukoudian M_1_). The EDJ of 9 specimens of *Au. africanus* (n = 1), *P. robustus* (n = 7), early *Homo* (n = 1) is remarkably simpler (Lei Pan pers. comm., see also Fig. 6) than those of Zhoukoudian, Hexian, and Yiyuan. Thirty-one M_2_s of European Early and Middle Pleistocene hominins and Neanderthals also display relatively simpler EDJ surface than those of *H. erectus* in China. In our sample of both fossil and recent *H. sapiens* (n = 63) (Fig. 6 and SI Fig. 14), we have not found any “dendrite-like” EDJ surface. Another two M_2_s from Xichuan of southern China, temporarily assigned to *H. erectus*, also exhibit a much crenulated EDJ surface with the development of several accessory ridges. A newly reported M_2_ from Hualong Cave, southern China, displays a much simpler EDJ surface compared to those of Zhoukoudian, Hexian, Yiyuan, and Xichuan. *Pongo* teeth are well known for having a complicated OES surface. However, the degree of complexity of OES is not reflected at the EDJ surface (SI Fig. 15). From the 18 fossil *Pongo* M_2_s observed in this study, we did not find any specimen with a dendrite-like EDJ like those of the *H. erectus* specimens mentioned above. The EDJ surfaces of some *Pongo* M_2_s are indeed quite smooth and in those cases when they are more crenulated the accessory ridges are generally thinner and lower than those from Zhoukoudian, Hexian, Yiyuan, and Xichuan. None of the hominins available in the literature show this type of highly-crenulated EDJ^38–41,45^. In these specimens, the secondary grooves and ridges of both the enamel and the dentine surfaces are also reflected at the occlusal surface of the pulp cavity, being a peculiarity not recorded in any other hominin group so far (Fig. 4).

At the EDJ, the protostylid-protoconid shelf combination, as in PA69, also exist in PA70 and Hexian M_2_ (PA839)^30^. Comparatively, the protostylid is less elevated in the samples of North African Middle Pleistocene^41^ and specimens of Sangiran Bapang-AG assemblage^40^.The PA70 root consists of two radicals that coalesce along the whole length and that do not narrow until the tip (SI Fig. 5). The 3D virtual reconstruction of PA70 in the present study reveals an enlarged pulp cavity (Figs 2 and 4) compared to that of ZKD KI and GI^4^, Hexian PA831 (Fig. 4)^46^ and Tighenif specimens^41^. Furthermore, the distal component of the coronal part of the pulp cavity in Zhoukoudian PA70, Hexian PA831, and Yiyuan Sh.y.072 is shallow due to the less elevated cuspal area (Fig. 4). This pattern is different from that of Hualong Cave specimen, Tighenif 1 and 2^41^ and NG92 D6 ZE 57 s/d 76 of Sangiran^40^, where the cuspal areas of the talonid are relatively sharper than those of Zhoukoudian, Hexian, and Yiyuan.

## Discussion

This is the first time, since the publications of the Zhoukoudian teeth by Black in 1927^1^ and Weidenreich in 1937^4^, that new original data, including a detailed and comprehensive study of the endostructure of most Zhoukoudian teeth preserved to date, are provided. This paper also presents the first direct comparisons of original Zhoukoudian sample with a large sample of Early and Middle Pleistocene teeth from Asia (e.g., Xichuan and Hualong Cave) and Europe (the Gran Dolina-TD6 and Sima de los Huesos fossils from Atapuerca). The new evidence confirms the similarities of the Zhoukoudian sample with other East Asian Middle Pleistocene hominins such as Hexian^30,46^, Yiyuan^44^, and Xichuan, and allow us to define a characteristic dental pattern for the populations that inhabited China during the Middle Pleistocene and that are usually classified as classic *H. erectus*.

Externally, Zhoukoudian teeth show the dental features that have been proposed in previous studies as typical of East Asian Middle Pleistocene *H. erectus*^30,44,46^ such as i) moderately convex labial surfaces, *tuberculum dentale* in the shape of several finger-like prolongations and pronounced shoveling in upper central incisors, ii) bucco-lingually expanded crown outline in M_2_, iii) bucco-lingually expanded mesial cusps compared to the distal cusps in molars, iv) rare occurrence of middle trigonid crest, (v) robust “column-like” dental roots that only narrow at the tip, and (vi) shelf-like protostylid and mesial protoconid ridge at the EDJ. These features are also partially present in Java (this study and references^32,35^) except for the Bapang fossil reported by Zanolli (2013)^47^ which present remarkably more simplified external and internal morphology.

Thanks to the application of micro-CT scanning this paper presents some morphological features at the dentine surface that have not been reported so far in any other hominin outside China and that could potentially represent unique characteristics of classic *H. erectus* in this region. Our study shows that the highly crenulated “dendrite-like” EDJ surface previously identified only in the Zhoukoudian, Yiyuan and Hexian M_2_s^30,44,46^, is also found in the M_1_ of Zhoukoudian, and the M_2_s of the Xichuan site from southern China. Surprisingly, the “dendrite-like” EDJ surface is also imprinted on the roof of the pulp cavity of these teeth. The crenulated labial surface of Zhoukoudian I^1^ might also be related to the complexity of the OES and EDJ of posterior teeth. To date, these features have been only described in Zhoukoudian, Yiyuan, Hexian and Xichuan hominins, and are absent in other hominin groups (*Australopithecus*, *Paranthropus* and *Homo)* analyzed by ourselves and/or available in the literature^38–41,48,49^. Unfortunately, no virtual reconstruction of the EDJ surface of Trinil teeth (holotype of *H. erectus*) is available (but see SI Fig. 16 for the relatively smooth EDJ lines of Trinil M^3^), so we cannot confirm whether this pattern is tentatively autopomorphic of the *H. erectus* taxon or a particularity of the *H. erectus* populations from China. In both cases, the morphological information provided here could be particularly useful to dentally define *H. erectus* from China and to distinguish them from other hominin lineages that may have potentially inhabited the continent at the same time.

Future studies and more data could shed light on the evolutionary meaning of this highly-crenulated EDJ surface. *H. erectus* from both continental and Southeast Asia has been characterized as displaying some degree dentognathic reduction in comparison to contemporaneous populations from Africa^43,50^. The dental reduction in these groups is assessed on the relatively smaller crown dimensions. However, the roots remain particularly large and robust, with a characteristic “column-like” aspect that conform the *H. erectus* dental bauplan we defined before. This pattern reflects the different ontogenetic mechanisms regulating the enamel and the dentine (e.g.^51–53^). The complicated conformation of the dentine could be related to the high mitotic activity and over-folding of the dentine surface in a relatively small crown. The high proliferative activity of the dentine could have a reflection in the general robusticity of the *H. erectus* skeletal remains and dental roots. Alternatively, an increasingly wrinkled surface may provide a functional adaptation to heavy occlusal attrition by increasing the length of the dentine surface. In the same line, the taurodontism found by Weidenreich (1937)^4^ in the Zhoukoudian specimens, and virtually reconstructed for the first time in the present study has been proposed as an adaptation to heavy occlusal attrition^54–56^. One hypothesis about the selective advantage of taurodontism is that a larger pulp cavity would allow for the deposition of secondary dentine and extend the longevity of a taurodont tooth^54–56^. An alternative hypothesis is that a more apical location of the bifurcation point would prevent its early exposure into the oral cavity, where it would be more easily affected by periodontal disease^56^. As dental attrition advances, the reduced height of the tooth would be compensated by the mechanism of compensatory eruption through apposition of cementum and remodeling of the alveolar socket^56,57^. In addition, the distal component of the coronal part of the pulp cavity is very shallow due to the less elevated cuspal area. Functionally, this might enlarge the dentine volume. Overall, the crenulated EDJ surface, the stout roots and the taurodontism could be mechanism favoring a tooth under high biomechanical demands despite a general dentognathic reduction, particularly of the crowns, in these populations^43,50,58^.

## Materials and Methods

### Materials

The present study focuses on the six isolated teeth of the Zhoukoudian *H. erectus* recovered from Zhoukoudian Locality 1 in 1950 s and 1960 s^28,29^. These teeth include I^1^ (PA66), P^3^ (PA67), P^4^ (PA68), P_3_ (PA110), M_1_ (PA69), and M_2_ (PA70) (See SI Table 4). PA66, 67, 68, 69, and PA70 were found in 1949–1959, and PA110 was recovered in 1966^28,29^. According to the excavation information provided in the primary report of these materials^28,29^, the six teeth can be assigned to their natural layers (SI Table 5), and their geological ages ranges from 230 kyr to > = 750 kyr according to different methods of chronometric analyses.

To be consistent with the aim of this study, the Zhoukoudian fossil teeth were compared to *H. erectus s.l*. from China, Dmanisi, Africa and Indonesia. And in order to better explore the polarity of the observed morphologies, a large sample of *Homo* from Africa, Asia and Europe was included, including recent modern humans that were sourced from Henan and Hubei Province (Central China) that span from the Neolithic to the Qing Dynasty times (SI Table 6–8).

Grouping of the comparative samples is mainly based on the geographic locations and geological ages^20,30,44^. For a detailed list see SI Table 6, 7, and 8. In addition to the Hexian and Yiyuan samples, we added Xichuan and Hualong Cave^59,60^ into the East Asian *H. erectus s.l*. comparative sample for the assessment of the EDJ surface (see SI Table 9 for the background information of these two sites).

### Methods

#### Grading occlusal wears and non-metrics

Tooth wear stages were scored according to the grading system by Molnar (1971)^61^. The dental terminologies used in the morphological descriptions and comparisons were cited from Weidenreich (1937)^4^, Turner *et al*. (1991)^62^, Scott and Turner (1997)^63^, and Martinón-Torres *et al*. (2008)^33^. Some of the non-metric were scored following the Arizona State University Dental Anthropology System (ASUDAS)^62^.

#### Linear metrics

Mesiodistal (MD) and buccolingual (BL) dimensions of the crown are taken from the primary report by Wu and Chia (1954)^28^, and Qiu *et al*. (1973)^29^. To investigate the crown outline shape of the molars, the crown index, calculated as BL*100/MD, was provided.

#### Microcomputed tomography and enamel-dentine junction surface reconstruction

To maximally extract morphological information of the Zhoukoudian teeth, each tooth was scanned using a 225 kV-μCT scanner (designed by the Institute of High Energy Physics, Chinese Academy of Sciences, and housed at the Institute of Vertebrate Paleontology and Paleoanthropology, Chinese Academy of Sciences) equipped with a 1.0-mm aluminum filter under settings of 120 kV, 100 uA, 0.5 rotation step, 360 degrees of rotation, 4 frames averaging (four times of scanning for each angle, and the four raw projections were coalesced). Isometric voxel size is 15.68–20.39 microns. Mimics 17.0 was used to complete the segmentation or virtual reconstruction of the EDJ surface and pulp cavity.

#### Geometric morphometric (GM) analysis

Geometric morphometric analysis was carried out on standardized occlusal surface pictures of P_3_ and M_2_ to examine the crown outline shapes. Only P_3_ and M_2_ were included into GM analysis based on the following considerations: 1) the crown of ZKD M_1_ is incomplete; 2) similar GM analyses have been carried out on P^3^ and P^4^ of most East Asian *H. erectus*^44^. However, the shapes of P^3^ and P^4^ will be referred whenever necessary in the comparison and interpretation of results. The details about how the photographs were taken, can be found in Xing *et al*.^44^. For a detailed explanation of the GM methods we refer to Zelditch *et al*.^64^.

For P_3_s, the anterior and posterior foveae were chosen as the landmarks. The apices of buccal and lingual cusps were worn in some of Zhoukoudian and other comparative specimens, and therefore not included into the GM analysis. The crown outline was equidistantly divided into 40 parts and the dividing points were semi-landmarks. Overall 42 landmarks and semi-landmarks were defined.

For M_2_s, the two intersection points between the crown outline and the mesiobuccal and lingual grooves were chosen as landmarks. The outlines of the trigonid and talonid were divided equally, using the TpsDig2 program^65^, into twenty parts. The division follows the principle that each part of the crown outline was roughly equal in length. The dividing points were treated as semi-landmarks. In total 40 landmarks and semi-landmarks were defined.

The TpsDig2 program^65^ was employed to digitize landmarks and semi-landmarks. The TpsRelw program^66^ was used to undertake superimposition on the raw coordinate data and the relative warp analysis (or principal component analysis) of shape variables.

### Availability of materials and data

All data generated or analyzed during this study are included in this published article (and its Supplementary Information files).

## Electronic supplementary material

Supplementary Information
