# Supplementary material for: The fossil teeth of the Peking Man
Source: Sci Rep. 2018 Feb 1;8:2066. doi: 10.1038/s41598-018-20432-y (PMC5794973; doi:10.1038/s41598-018-20432-y)
Supplement: Supplementary file 1 — Supplementary Information [file 41598_2018_20432_MOESM1_ESM.docx]

**The fossil teeth of the Peking Man-**Supplementary information

Song Xing^a*^, María Martinón-Torres^b,c^, José María Bermúdez de Castro^b,c^

*a Key Laboratory of Vertebrate Evolution and Human Origins of Chinese Academy of Sciences, Institute of Vertebrate Paleontology and Paleoanthropology, Chinese Academy of Sciences, Beijing 100044, China*

*b Centro Nacional de Investigación sobre la Evolución Humana (CENIEH), Paseo de la Sierra de Atapuerca s/n, 09002 Burgos, Spain*

*c University College London (UCL) Anthropology, 14 Taviton Street, London WC1H 0BW, UK*

**SI Text**

***Description of dental morphologies***

**Left upper central incisor (I^1^ PA66)**

This tooth has complete crown and root, and the occlusal wear corresponds to grade 3 of Molnar (1971)^1^ (SI Figure 1). The labial surface is moderately convex (ASUDAS grade 3) in mesial-distal direction viewed occlusally. In the lingual side, both mesial and distal marginal ridges are well-developed and define a shoveling of ASUDAS grade 4. The basal eminence is prominently bulged and five finger-like projections of different sizes originate from it (tuberculum dentale grade 4). There is also a lingual central ridge that reaches the incisal edge and is also reflected in the labial surface. The labial surface of the crown is furrowed by seven longitudinal grooves. The cervical line in the buccal aspect is approximately straight. The root is wide and stout, buccolingually compressed. Viewed laterally, the root shrinks abruptly to a small tip from its middle length.

The shoveling, finger-like lingual projections, and the labial furrows on the enamel surface are also reflected at the EDJ surface (Figure 2 and 3). The coronal part of the pulp cavity bifurcates into a mesial and a distal branch place before reaching the incisal end.

**Right upper third premolar (P^3^, PA67)**

This tooth is complete with just a small enamel crack in the lingual cusp (SI Figure 1). Its occlusal wear is at grade 3 of Molnar (1971)^1^ with a small dot of dentine exposure in the buccal cusp. From the occlusal view, the crown contour is oval and asymmetrical. The buccal and lingual cusps are connected by a continuous transverse crest. A secondary groove bifurcates the essential crest of the buccal cusp. On the buccal surface, there is a pronounced central ridge well delimited by a moderate and weak mesial and distal depressions, respectively. The buccal surface presents several short wrinkles. The root is composed of two broad radicals that separate from the second third of the root but are joined by a mesial lamina of dentine/cementum. A longitudinal furrow is present in the distal aspect of the buccal radical. Viewed laterally, the breadth of the root does not reduce until the very apical end.

All the features described on the enamel surface are found at the EDJ (Figure 2). In addition, the mesial and distal accessory ridges of the lingual cusp can be identified at the dentine. There are a buccal and lingual radicals that separate from at a third of the root length. The canal of the buccal radical is further bifurcated into two branches in its apical third.

**Right upper forth premolar (P^4^, PA68)**

This tooth is complete with a grade 3 of occlusal wear degree (Molnar, 1971)^1^ (SI Figure 1). The crown contour is ellipse-like and slightly asymmetrical, and the widths of the buccal and lingual cusps are roughly equal. The essential crest of the buccal cusp is bifurcated, with the mesial branch deflects and connects with the mesial marginal ridge whereas the distal branch connects with the lingual cusp and conforms a middle continuous transverse crest. Mesial and distal accessory ridges for both buccal and lingual cusps can be identified. On the buccal surface, the central ridge is delimited by weak mesial and distal vertical grooves. The root is wide and composed of two radicals that coalesce along most of its length except for a strongly bifurcated tip. The buccal and lingual root radicals are marked by wide longitudinal grooves on both mesial and distal sides. From the lateral view, the root width does not decrease until its apical third.

All the features described at the enamel surface can be identified at the EDJ, and the several crests and wrinkles provide a complex EDJ surface (Figure 2). Although the root canal is single, the pulp cavity is large and wide and the tooth can be classified as hypertaurodont (Figure 4).

**Right lower third premolar (P_3_, PA110)**

This tooth has a complete crown, but the apical forth and a large mesiobuccal aspect of the root are missing (SI Figure 5). The occlusal wear corresponds to grade 3 of Molnar (1971)^1^. The crown contour is asymmetrical with a slightly protruding distolingual corner. The mesiolingual cusp (the metaconid) is weakly developed and the apex of the lingual cusp was worn. There are two continuous transverse crests and, mesially bounded by a linear-like and open anterior fovea and distally, by a pit-like fovea. On the buccal surface, both mesial and distal vertical grooves are moderately developed. Although incomplete, the root can be reasonably estimated to be single with weakly-developed mesial and distal longitudinal grooves (ASUDAS Tomes’ root grade 0).

At the EDJ surface, the apex of lingual cusp can be seen (Figure 2). The transverse crest is divided into two sharp ridges by a deep groove. On the buccal surface, the mesial vertical groove is more pronounced than the distal one, and they delimit mesial and distal buccal marginal ridges. The root canal is single, confirm the Tomes’ root structure of ASUDAS grade 0.

**Left lower first molar (M_1_, PA69)**

This tooth is complete except for a fragment of enamel and dentine in the lingual aspect (SI Figure 5). The crown and the root present several cracks. The wear degree corresponds to grade 3 of Molnar (1971)^1^.

With the estimation of the missing part, the crown of PA69 is approximately rectangular, with the trigonid and talonid showing a similar width. The five main cusps are present and arranged in a “Y” pattern. The hypoconulid is large (grade 4 of ASUDAS). No middle or distal trigonid crests are expressed. A grade 2 (ASUDAS) of defecting wrinkle can be scored on the metaconid. Due to the occlusal wear and breakage, the absence or presence of a C6 and C7 cannot be confirmed. Root is composed by a mesial and a distal radical well bifurcated from the second third and that run parallel towards the tip. Longitudinal depression run at the mesial and distal surface and a bifid tip can be seen at the end of the mesial root. From a lateral view, the width of the roots does not decrease clearly until the very apical end. On the buccal surface, the protostylid is represented by a shallow and short groove (ASUDAS grade 3 or 4). Another moderately-developed groove is present mesial to the protostylid.

The EDJ is remarkably crenulated, with several grooves and ridges which bifurcate and intersect with each other and conform a dendrite-like pattern (Figure 2). The C6 is not present and a large and linear distal fovea can be found. On the buccal surface, a triangle-like dentine elevation without free apex corresponds with the protostylid. Mesial to the protostylid there is a moderately-developed groove, that can be also identified at the enamel and that define a protoconid shelf^2^. The root is composed of three canals. The mesial and distal root canals have a high bifurcation and the mesial one further bifurcates into a buccal and a lingual one from the apical third.

**Left lower second molar (M_2_, PA70)**

This tooth has complete crown and root (SI Figure 5), the occlusal wear corresponds to grade 2 of Molnar scoring system (1971)^1^. The crown contour is approximately oval. The hypoconulid is well developed (grade 4 of ASUDAS) and buccally displaced. The C6 is delineated by two secondary grooves in the distal end of the crown and is much smaller than the hypoconulid (grade 1 of ASUDAS). No C7 is identified. The five main cusps are arranged in a “Y” shape. The essential crests of both protoconid and metaconid are connected with the mesial marginal ridge with continuous mesial trigonid crest. The occlusal surface is complicated by developments of secondary grooves and ridges. On the buccal surface, the protostylid is represented by a shallow and short groove (ASUDAS grade 3 or 4). Another moderately-developed groove is present mesial to the protostylid. The root consists of two radicals that coalesce along the whole length and that do not narrow until the tip.

The cusp and groove patterns on the enamel surface matched those at the EDJ (Figure 2). The presence of multiple grooves and ridges that intersect with each other compose a remarkably complex dendrite-like EDJ surface. The protostylid is more developed at EDJ, and represented by a moderate dentine crenulation. Mesial to the protostylid there is a protoconid shelf (see reference^2^). The occlusal surface of the pulp cavity is generally flat except for the two elevated mesial dentine horns. The imprint of secondary grooves and ridges typical of both the enamel and dentine surfaces is reflected at the occlusal surface of the pulp cavity (Figure 4). There is a large pulp cavity that does not bifurcate until the second third so the tooth can be classified as mesotaurodont.

***Comparative dental morphology***

**Upper central incisor (I^1^)**

Although variable in recent modern human^3^, the degree of shoveling increases from earlier hominins (*Australopithecus* and early *Homo*) to Eurasian Middle Pleistocene hominins and Neanderthals^4^. The pronounced shoveling found in Zhoukoudian (SI Figure 1 and 2) could also be identified in Hexian (PA835), and is more developed than in African *H. ergaster* (KNM-WT 15000 and KNM-ER 803), Dmanisi (D2736), and Sangiran hominins^5-7^.

Labial surface is generally weakly convex mesiodistally in earlier hominins, and reach its maximum degree in European Middle Pleistocene hominins and Neanderthals^4,7,8^. The convexity of labial surface in Zhoukoudian (SI Figure 1 and 2), is similar to that of Xujiayao^9^, can be encompassed within the variation of the Sangiran specimens from Grenzbank/Sangiran assemblage (where it range from faint to strong) and is more pronounced than that of the Dmanisi D2736^7^ and Hexian PA835^10^. Sangiran S7-1 from the later formation of Sangiran (Bapang-AG assemblage) is moderately convex in the buccal surface^6^.

Zhoukoudian I^1^s displays a well-defined and pronouncedly bulged basal eminence as it is typically recorded in the Middle Pleistocene populations from Europe and Neanderthals^8^. A well-developed basal eminence can be also seen in the I^1^s from Dmanisi^7^, Sangiran S7-1, Sangiran S7-48^6^, and Panxian Dadong^11^, but not in the Sangiran S7-85 and S7-86^6^. In addition, Zhoukoudian I^1^s express a number of finger-like prolongations in the lingual aspect, similar to those found in KNM-WT 15000^12^, some Neanderthals like those from Krapina. Panxian Dadong I^1^ from Chinese late Middle Pleistocene is similar as Zhoukoudian in the existence of pronounced basal eminence, but differs in less numbers of finger-like prolongations^11^. Modern human is much less developed in the lingual basal eminence and numbers of finger-like prolongations (0-3) related to Zhoukoudian.

Lingual central ridge detected in PA66, ZKD 2, and ZKD 4 is a rare trait that can only be found in KNM-ER 803^5^, Sangiran S7-86/7-86^6^, Yuanmou, and a very few specimens (6.1%) of recent modern human among the specimens involved in the present study. Comparatively, the lingual central ridge of Zhoukoudian I^1^ is less elevated than those of other *H. erectus sensu lato*. The buccal enamel wrinkles and lingual finger-like prolongations found at the outer enamel surface of Zhoukoudian and Hexian I^1^s can be matched at the EDJ (Figure 3), as well as at the surface of pulp cavity (Figure 4). Similar wrinkle features could also be observed in outer enamel surface of *Australopithecus*, and probably represent a primitive feature. However, no images of the EDJ at this level are available for *Australopithecus* so the taxonomic value of this feature needs further exploration. The lateral view of the pulp cavity of Zhoukoudian and Hexian I^1^s show a bulged canal in the lingual aspect, and this might correspond with the stout outer looking of the root (Figure 4).

**Upper third premolar (P^3^)**

The occurrence of a transverse crest, as in PA67 (SI Figure 1), is more frequent in *Australopithecus* and early *Homo* groups^5,13^. Its frequency decreases in later *Homo* groups although present in Sangiran 4, Sangiran 7-35, Hexian PA832, ZKD16^6,10,14^. It can be also found in KNM-ER 3733 and in 6 out 19 specimens from Sima de los Huesos^8^. P^3^s from Panxian Dadong and Xujiayao do not express a transverse crest as in Zhoukoudian PA67^9,11^.

Although PA67 has coalesced buccal and lingual roots with bifid apices when observed outside, it has three independent root canals based on the 3D reconstruction and should be classified as three-rooted type (Figure 2 and 4). ZKD 77 and 78 also has its buccal and lingual roots combined, while the two roots are completely separated and divergent from each other in ZKD 19^14^. Among the *H. erectus* *sensu lato*, coalescence of buccal and lingual roots could also be found in D3672 of Dmanisi and KNM-WT 15000^7,12^, while the rest of P^3^s observed in the present study commonly have two independent roots^5,6,15^. In a few cases (Hexian PA832, Sangiran 7-35/7-36, and KNM-ER 1808), a third radical can be identified^5,6,10^.

**Upper forth premolars (P^4^)**

P^4^s of *Australopithecus* and early *Homo* tend to have a slightly asymmetrical crown outline with the lingual cusp being wider than the buccal cusp^15^, and this type of shape could also found in D2282 from Dmanisi^7^. Comparatively, other members of *H. erectus* *sensu lato* (Sangiran, Yiyuan, KNM-WT 15000, and KNM-ER 3733) generally have its buccal and lingual cusps being approximately same in the width. In the later *H. sapiens*, there is a tendency to have a wider buccal cusp than the lingual cusp^15^.

The presence of a continuous transverse crest, like that in PA68, can also observed in ZKD27 from Zhoukoudian Locality 1^14^, but absent in ZKD 25 and ZKD 133’ (SI Figure 4). This feature could also be observed in *Australopithecus*, early *Homo*, and other samples of early *H. erectus* *sensu lato* (e.g. Dmanisi D2282, Sangiran 7-29, and KNM-ER 3733)^5-7,13^, either in continuous or interrupted form. It is not present in other *H. erectus* *sensu lato* from East Asian mid-Middle Pleistocene^15^. 4 out of 19 from Sima de los Huesos were documented to have interrupted transverse crest in Martinón-Torres et al. (2012)^8^, and apart from this, Neanderthals and *H. sapiens* have a very low frequency of transverse crest^8^.

*Australopithecus* and early *Homo* are characterized by two or three independent roots^5,13^, although the early *Homo* OH 16 presents two coalesced roots^16^. The number of premolar roots in *H. erectus* *sensu lato* ranges from three to two, and in some cases the buccal and lingual roots are coalesced^5-7^. The root structure as shown in PA68 is similar to ZKD28, ZKD 87 (SI Figure 4), and Yiyuan Sh.y. 007^14,15^. Sangiran 17^17^ and Sangiran S7-37^6^ still remain the independently separated buccal and lingual roots. Taurodontism was commonly documented in molars (e.g., Shaw, 1928^18^; Kupczik and Hublin, 2010^19^), but also found in a very low frequency in premolars^20-23^. The enlarged pulp cavity (hypertaurodont; this terminology is cited from Shaw [1928]^18^), as shown in the Zhoukoudian PA68, was also observed in another East Asian Middle Pleistocene hominin from Yiyuan (Figure 4).

**Lower third premolar (P_3_)**

Compared with modern human and European Middle Pleistocene hominins and Neanderthals, *Australopithecus* and early *Homo* tend to have more asymmetrical crown outlines, buccolingually more elongated, and with widely-separated anterior and posterior foveae (SI Figure 6). The occlusal polygon is also mesially displaced polygon due to the presence of a conspicuous talonid. Zhoukoudian specimens cluster with most specimens of Sangiran, Tighenif, TD6, KNM-WT 15000, and KNM-ER 992, apart from the *Australopithecus* and Early *Homo* (SI Figure 6). D211 and D2375 from Dmanisi tend to have a more mesiodistally elongated and asymmetrical crown outline than Zhoukoudian specimen, and group with early *Homo*. Compared to Zhoukoudian, the crown outline of S9 from Sangiran is more buccolingually elongated. Among the Zhoukoudian specimens, PA110, ZKD20, ZKD80, and ZKD Z are distributed close to each other in the graphic with similar crown outline shape, while ZKD 81 and ZKD82 are more separated from the rest due to their buccolingually elongated crown outline.

The mesial and distal vertical grooves on the buccal surface of PA110 are both moderately developed. The mesial vertical groove in other Zhoukoudian specimens vary from being faintly (ZKD21) to be pronouncedly developed (ZKD82), while the degree of distal vertical groove ranges from absence (ZKD21) to moderate (ZKD80, 81, Z) (SI Figure 7). The buccal vertical grooves are well-developed in *Australopithecus*, usually defining a marginal enamel shelf. They are less pronounced in specimens of *H. erectus* *sensu lato* and variable among different individuals. These grooves vary from faint to strong in the Grenzbank/Sangiran assemblage, and varied from being faint to moderate in those from Bapang-AG assemblage^6,24^. D211 from Dmanisi has a pronouncedly developed vertical groove in the mesial side and weak vertical groove in the distal side^7^. Comparatively, D2735 is less developed in the buccal vertical groove (faintly or weakly developed)^7^. Comparatively, samples of European Middle Pleistocene hominins, Neanderthals, and Panxian Dadong generally have a smooth buccal surface^8,11^.

Talonid is generally well-developed in *Australopithecus* and early *Homo*, where it contains both buccal and lingual components^5,13^. Although relatively less developed, the talonid still involve both buccal and lingual aspects of the distal crown in Early Pleistocene members of *H. erectus sensu lat*o, and Zhoukoudian sample (e.g., ZKD 80, 81, 85, 130’, and Z)^6,7,14,24^. However, ZKD 20 does not display a talonid (SI Figure 7).

The root is robust, but it tends to simplify in PA110, ZKD21, ZKD23, ZKD82 and ZKD85^14^ (See also SI Figure 7). PA110, ZKD21, and ZKD23 have a single root, while ZKD82 and 85 have a Tomes’ root with two mesial and one distal longitudinal grooves (SI Figure 7). In both Tighenif 1 and Tighenif 2 P_3_s, the root structure is composed of three fused radicals^25^ as in Zhoukoudian 82 and 85 (SI Figure 7). This root pattern can be also observed in Trinil 5 (personal observation), Chenjiawo mandible from Lantian (unpublished data, micro-CT scanning), D211 of Dmanisi^26^, Sangiran 22^24^, and probably in Sangiran 6^24^. Apart from the single-rooted Tomes’ pattern, a more complex root structure can be observed in other members of *H. erectus* *sensu lato* from Dmanisi, East Africa, and Sangiran^5,7,24^. If we follow Wood et al. (1988)^27^, D2375 from Dmanisi and KNM-ER 992 are classified as 2T, Sangiran 8 and 9 and KNM-ER 730 as 2R: MB+D^27^, 2R: M+D in D2600. A more complex root structure is more commonly found in earlier hominins including *Australopithecus* and early *Homo*^13,27^.

**Lower first molar (M_1_)**

As shown in SI Figure 9, the crown index indicates that Zhoukoudian M_1_s, similar to those from Sangiran and Tighenif, tends to have a relatively wide crown (but see Sangiran 6). Comparatively, the *H. ergaster* specimens from East Africa and Dmanisi resemble *H. habilis* (referred as early *Homo* in this study)^28^ in having a relatively mesiodistally elongated crown outline. The independent t-Test indicated that the crown index of Zhoukoudian is significantly higher than African early *Homo* or *H. ergaster* (SI Table 2). *Australopithecus* overlap with all members of *H. erectus sensu lato* in a wide range. The median value of M_1_ crown index in European Middle Pleistocene, Neanderthals and recent *H. sapiens* is closer to East Asian members of *H. erectus* *sensu lato* than to those from East Africa and Dmanisi. The Zhoukoudian M_1_s are characterized by having a crown outline that is wider in the mesial aspect (more buccolingually elongated trigonid than the talonid). The trigonid and talonid in *Australopithecus* are generally of the same width, but the talonid is slightly wider in a few cases like Stw 246 and Stw 309. Similar to early *Homo*, other members of *H. erectus* *sensu lato* from Africa, Dmanisi, and Sangiran tend to have trigonids and talonids of the same width. In other later *Homo* specimens including recent modern human, there is a tendency to have a wider talonid than the trigonid. Summarizing, the expression of a talonid that is narrower than the trigonid in 5 out of 9 Zhoukoudian teeth suggests that this feature, although not exclusive to this population (e.g. Sangiran 22^24^ and Dmanisi D211^7^) seems to be a particularity of the Zhoukoudian hominins with regard to other *H. erectus sensu lato*.

Trigonid crest are uncommon in the Zhoukoudian M_1_s sample. We have only identified a continuous mesial trigonid crest (MeTC) in ZKD34 (SI Figure 8). Based on the frequencies scored on the EDJ surface, this type of MeTC is only found in a very small numbers of Neanderthals (2 out of 16) and *H. sapiens* (1 out of 12)^29^. None of the Zhoukoudian M_1_s (SI Figure 8) have a continuous middle trigonid crest (MTC). This trait is usually found in a high rate in European Middle Pleistocene hominins and Neanderthals^8,30^. It can be also found in *Australopithecus* (9 out of 25), Dmanisi (D211 and D2735), and Sangiran specimens from both Grenzbank/Sangiran assemblage (e.g. Sangiran 6, Sangiran S7-76) and Bapang-AG assemblage (SB 8103), and is rarely present in samples of early *Homo* and African *H. ergaster*, as well as the *H. sapiens*^6-8,13,24^.

As in PA69, ridge-like protostylid delineated by secondary grooves on the buccal surface can also be observed in ZKD 36 and 43. Comparatively, ZKD97, 98, 137’ have shelf-like protostylid (SI Figure 8). The later form of protostylid also presents in Dmanisi (e.g. D211)^7^, Sangiran (e.g. Sangiran 6)^24^ and some *Australopithecus* (e.g., Stw 130, 309, 421)^13^.

The EDJ surface of Zhoukoudian PA69 is highly crenulated mainly due to the developments of several secondary ridges accompanying the essential crest of the cusps. Based on the EDJ reconstructions available in the literature, the degree of crenulation of Zhoukoudian PA69 exceeds that of *A. africanus* (e.g., Stw 309, 421) and *Paranthropus robustus* (e.g., SK104, 828)^31^, the late Early Pleistocene hominin from Mulhuli-Amo of East Africa (MA93)^32^, the North African Middle Pleistocene hominins from Tighenif (Tighenif 2)^25^, and other later *Homo*^29,33^. Although no virtual reconstruction of the EDJ surface of Trinil molars (holotype of *H. erectus*) is available, the slices from micro-CT scanning of two M^3^s display a relatively smooth EDJ line in those two specimens (data from ESRF) (SI Figure 16). The protostylid-protoconid shelf combination at the buccal surface of EDJ of Zhoukoudian PA69 is less obvious on the Tighenif 2^25^ and not present on MA93 from East African late Early Pleistocene^32^. Although this cingulum-like structure exists in other later *Homo* specimens^29,33^, its expression is weaker than that of Zhoukoudian PA69.

**Lower second molar (M_2_)**

Both the expression of a well-developed hypoconulid (from moderate to large size) and a C6 (6 out of 7 specimens) are common in the Zhoukoudian sample as in other members of *H. erectus* *sensu lato* and earlier hominins including *Australopithecus* and early *Homo*^5,13^. The C6 is less frequent in European Middle Pleistocene populations, Neanderthals, and *Homo sapiens*^8^. There is no C7 in the 7 specimens available from Zhoukoudian Locality 1^14^ (SI Figure 11) whereas this feature is comparatively more frequent in *H. ergaster,* Dmanisi and Sangiran hominins^5-7,24^.

A continuous MTC, typically found in European Middle Pleistocene hominins and Neanderthals^8,30^, can be also found in isolated specimens from African (KNM-ER 1808), Dmanisi (D2375), and Sangiran (S9), but it is absent in the Zhoukoudian sample^5,7,14,24^. Instead, PA70 of Zhoukoudian display a mesial trigonid crest (MeTC) (SI Figure 5). This MeTC was absent in the *H. sapiens* sample of Martínez de Pinillos et al.^29^ and although not found in the Neanderthal sample of Bailey et al. (2011)^33^, Martínez de Pinillos et al. (2014)^29^ reported an occurrence of 25.0% in Neanderthals and 27.2% in Sima de los Huesos. It could also be seen in one of the European Early Pleistocene specimen (ATD6-96). In addition, no *Australopithecus* has been documented to have MeTC^33^.

Protostylid is well-developed in Zhoukoudian specimens, and it can appear as a long shelf-like feature extending throughout the whole buccal surface (ZKD45 and 52) or as a short groove stemming out from the buccal groove (PA70)^14^ (SI Figure 11). The long shelf-like protostylid is usually found in *Australopithecus* (e.g., Stw 560 and MLD 2), and can be also found in some early *Homo* (OH16), Sangiran hominins from Grenzbank/Sangiran assemblage (S5) and Yiyuan hominins^13,15,16^.

The most remarkable feature of the Zhoukoudian PA70 is found at the EDJ (Figure 2 and 6). The EDJ surface of this tooth is as highly crenulated, with interconnected ridges, bifurcated essential crests, accessory ridges, accessory cusps like C6. This type of dendrite-like EDJ surface has been also found in Hexian, Yiyuan, and Xichuan molars^10,15^, and are more complicated than those from the European Middle Pleistocene, Neanderthals, *H. sapiens*^29,33,34^, and the Sangiran specimens from Bapang-AG assemblage reported by Zanolli^35^). Although available data on the EDJ surface from other hominins are limited, it seems that the Zhoukoudian M_2_ EDJ is also more crenulated than in some *Australopithecus* specimens^31^ and the Tighenif specimens^25^. They tend to be thinner and less elevated. In addition to the complexity in EDJ surface, to our knowledge this is the first time that the imprint of the profuse crenulation can be also found at the occlusal surface of the virtually reconstructed pulp cavity (see Zhoukoudian and Hexian M_2_s, Figure 4).

The protostylid-protoconid shelf combination at the buccal surface of EDJ of Zhoukoudian PA70 can be also found in one of the Hexian M_2_s (PA839)^10^. The protostylid of Tighenif 2 from North African Middle Pleistocene^25^ and NG0802.3 from Sangiran Bapang-AG assemblage^35^ is less elevated than that of Zhoukoudian PA70. In addition, the protoconid shelf is absent from NG0802.3 and faintly developed in Tighenif 2^25^. These features are also generally absent in the European Middle Pleistocene hominins, Neanderthals, and *H. sapiens*^29^.

Molar taurodontism is commonly documented in specimens of Neanderthal-lineage^19^ and also could be variably detected in modern human^18,36^. Taurodont molar was used to be reported in Zhoukoudian specimens (Zhoukoudian Mandible BI and KI) by Weidenreich (1937)^14^ and probably in African *H. ergaster* (e.g. KNM-ER 992)^5^ through an x-ray assessment. The 3D virtual reconstruction of PA70 in the present study reveals a more enlarged pulp cavity than those of ZKD KI and GI (Weidenreich, 1937), Hexian (Figure 4)^10,37^ and Tighenif specimens^25^. Furthermore, the distal component of the coronal part of the pulp cavity in Zhoukoudian, Hexian, Yiyuan, and Xichuan M_2_s is very shallow due to the less elevated cuspal area (Figure 4). This situation is different from Tighenif 1 and 2^25^ and NG92 D6 ZE 57s/d 76^35^, where the cuspal areas of the talonid are relatively sharper than those of Zhoukoudian, Hexian, and Yiyuan.


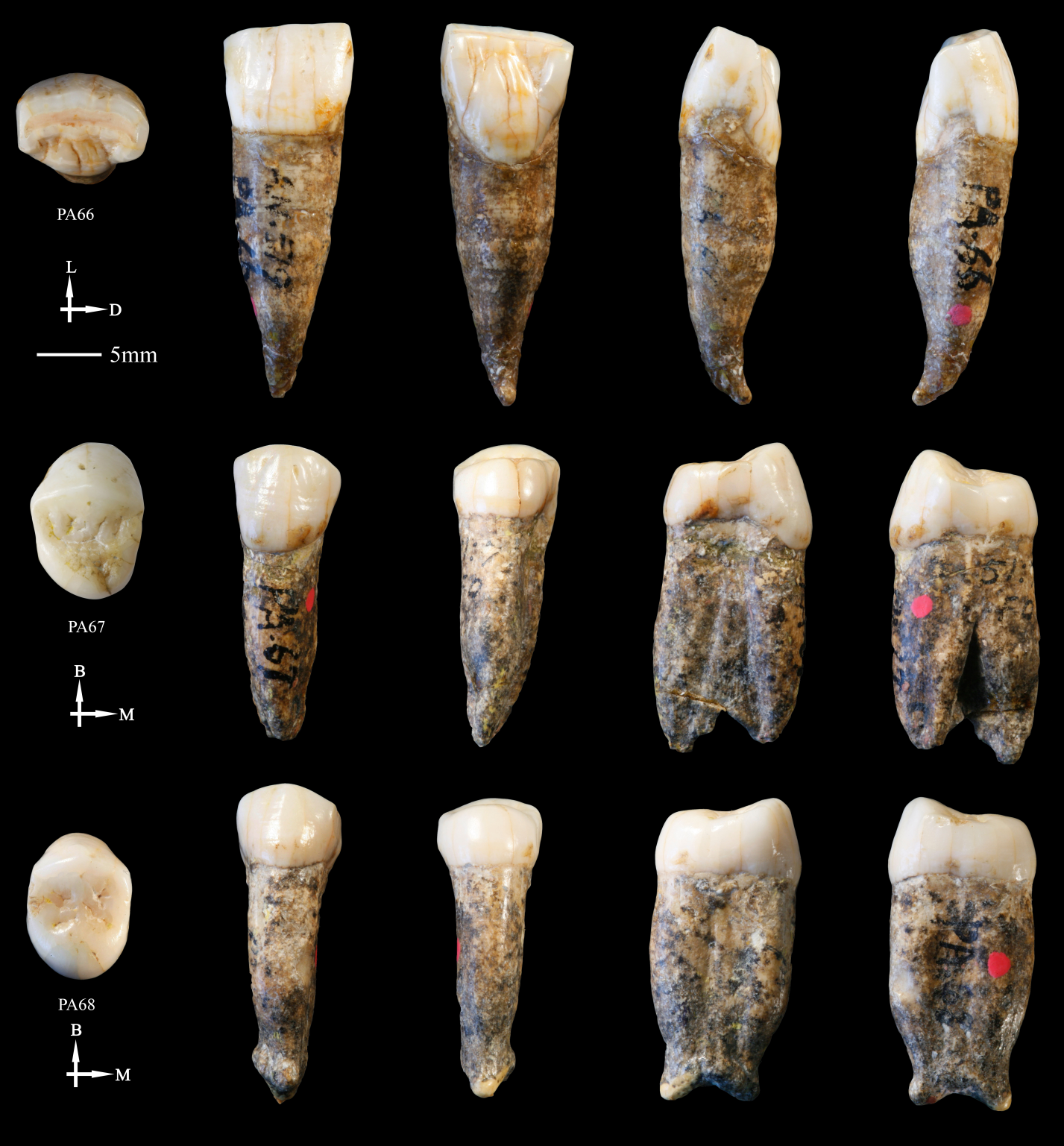


SI Figure 1. The maxillary teeth from Zhoukoudian Locality 1 (I^1^, P^3^, and P^4^). From left to right are occlusal, buccal/labial, lingual, mesial, and distal views. B: buccal, D: distal; L: lingual, M: mesial.


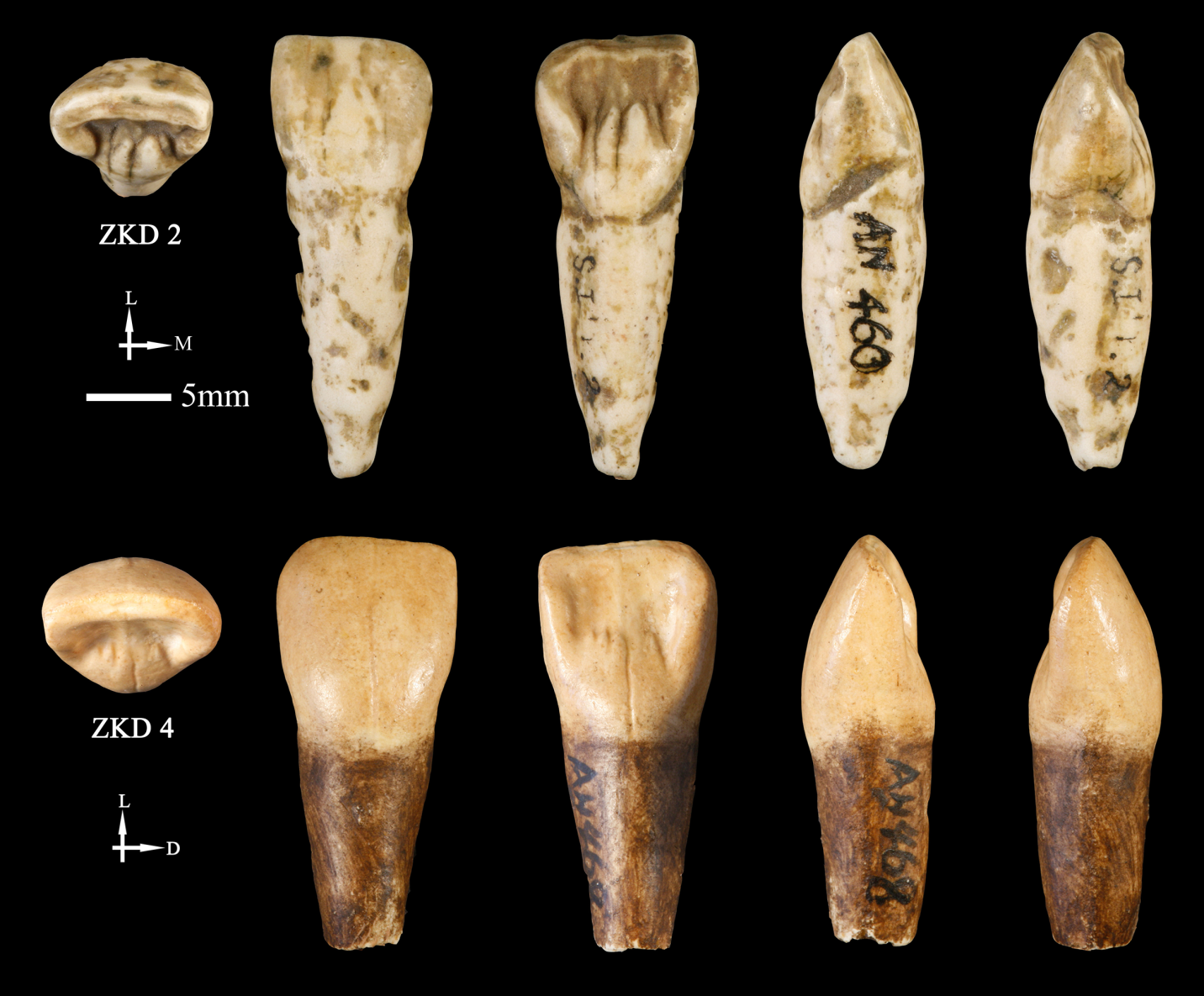


SI Figure 2. Upper central incisors of Zhoukoudian. From left to right are occlusal, labial, lingual, mesial, and distal views. D: distal; L: lingual, M: mesial.


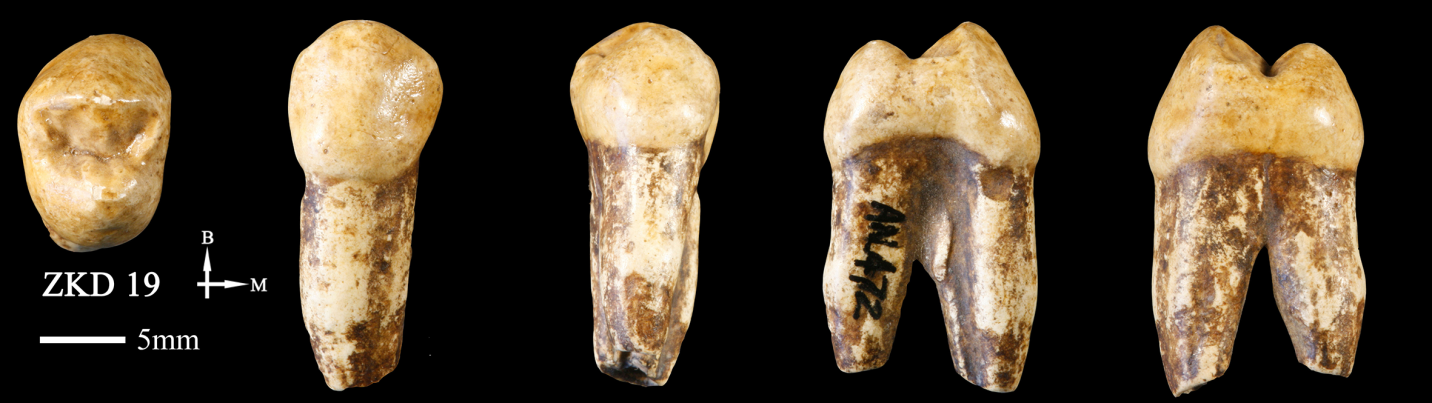


SI Figure 3. Upper third premolar of Zhoukoudian. From left to right are occlusal, buccal, lingual, mesial, and distal views. B: buccal, M: mesial.


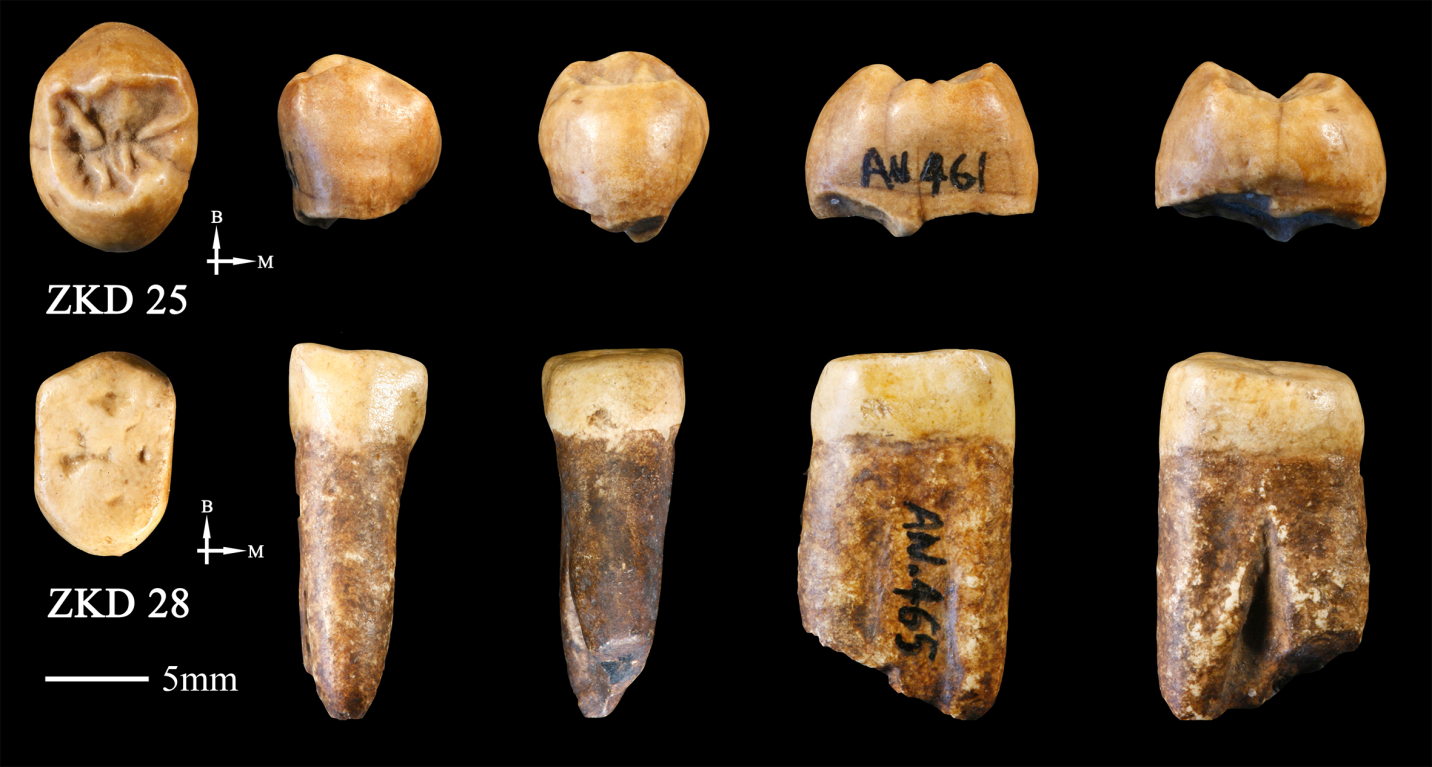


SI Figure 4. Upper forth premolars of Zhoukoudian. From left to right are occlusal, buccal, lingual, mesial, and distal views. B: buccal, M: mesial.


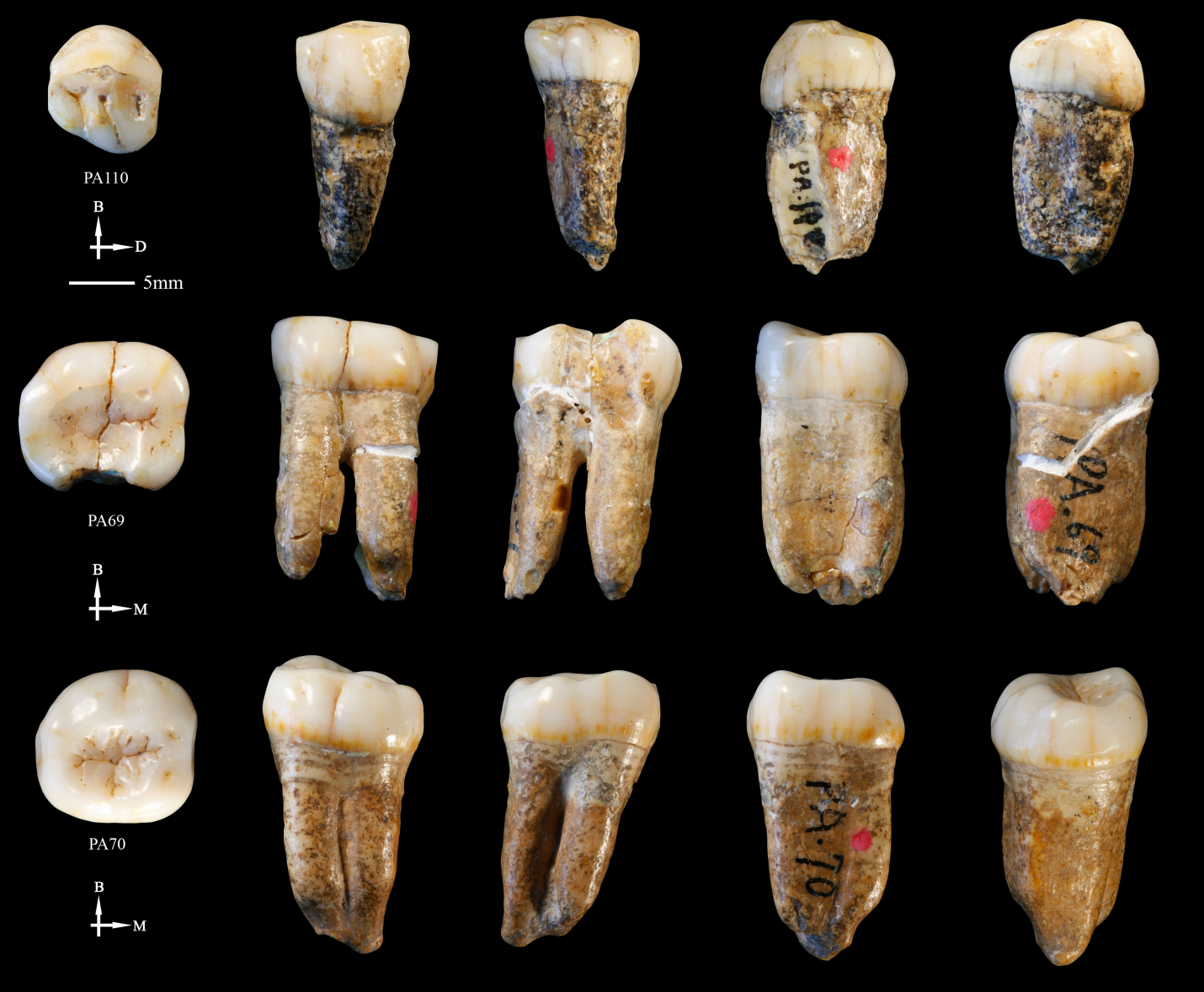


SI Figure 5. The mandibular teeth from Zhoukoudian Locality 1 (P_3_, M_1_, and M_2_). From left to right are occlusal, buccal, lingual, mesial, and distal views. B: buccal, D: distal, M: mesial.


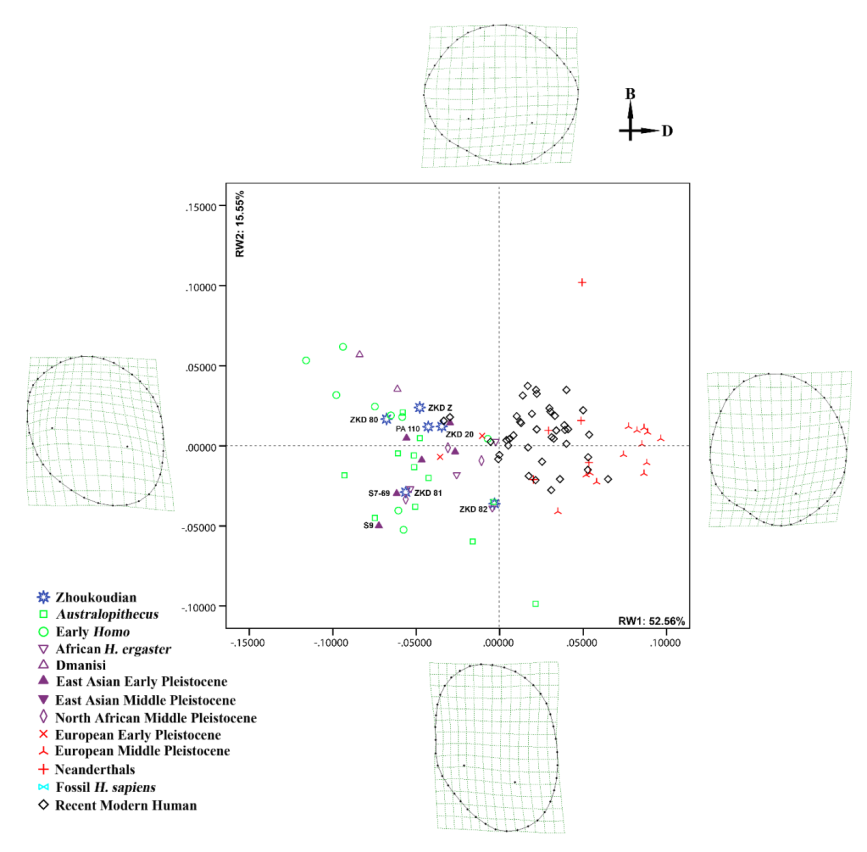


SI Figure 6. Geometric morphometric analyses of P_3_ crown outline shapes and the position of anterior and posterior foveae (TPS-grids correspond to the positive-value or negative-value extremes of the RW axis). B: buccal; D: distal.


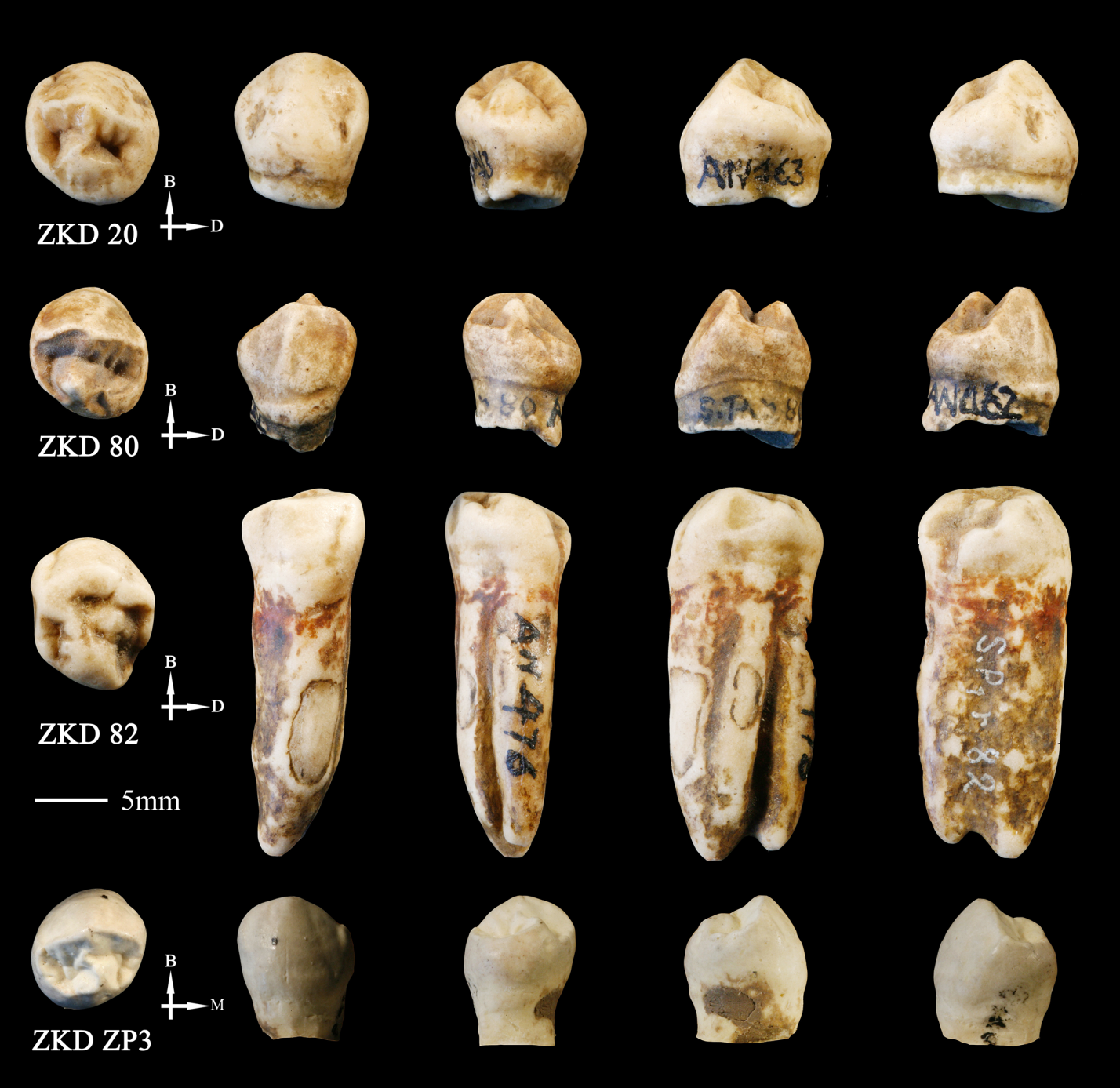


SI Figure 7. Lower third premolars of Zhoukoudian. From left to right are occlusal, buccal, lingual, mesial, and distal views. B: buccal, D: distal, M: mesial.


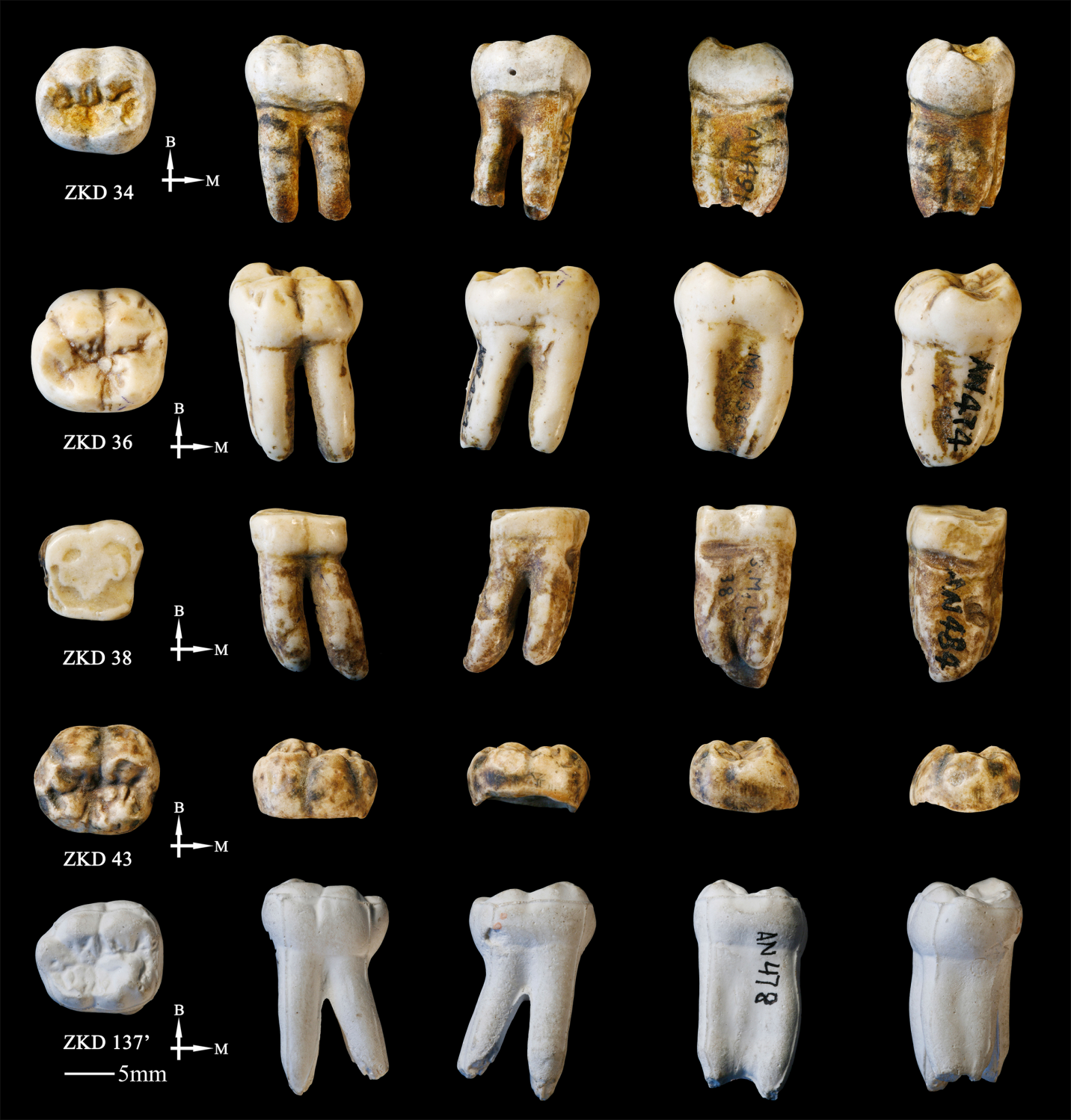


SI Figure 8. Lower first molars of Zhoukoudian. From left to right are occlusal, buccal, lingual, mesial, and distal views. B: buccal, M: mesial.


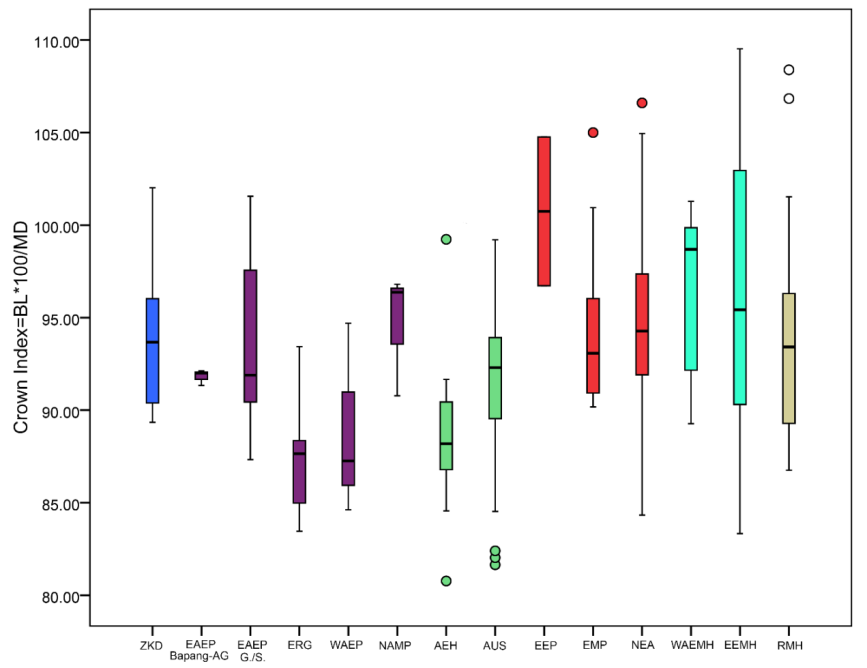


SI Figure 9. Boxplots of crown indices of Zhoukoudian M_1_s and other comparative specimens. ZKD: Zhoukoudian; EAEP Bapang-AG: East Asian Early Pleistocene-Sangiran Bapang-AG assemblage; EAEP G./S.: East Asian Early Pleistocene-Sangiran Grenzbank/Sangiran assemblage; ERG: *Homo* *ergaster*; WAEP: West Asian Early Pleistocene; NAMP: North African Middle Pleistocene; AEH: African early *Homo*; AUS: *Australopithecus*; EEP: European Early Pleistocene; EMP: European Middle Pleistocene; NEA: Neanderthals; WAEMH: West Asian early modern human; EEMH: European early modern human; RMH: Recent modern human.


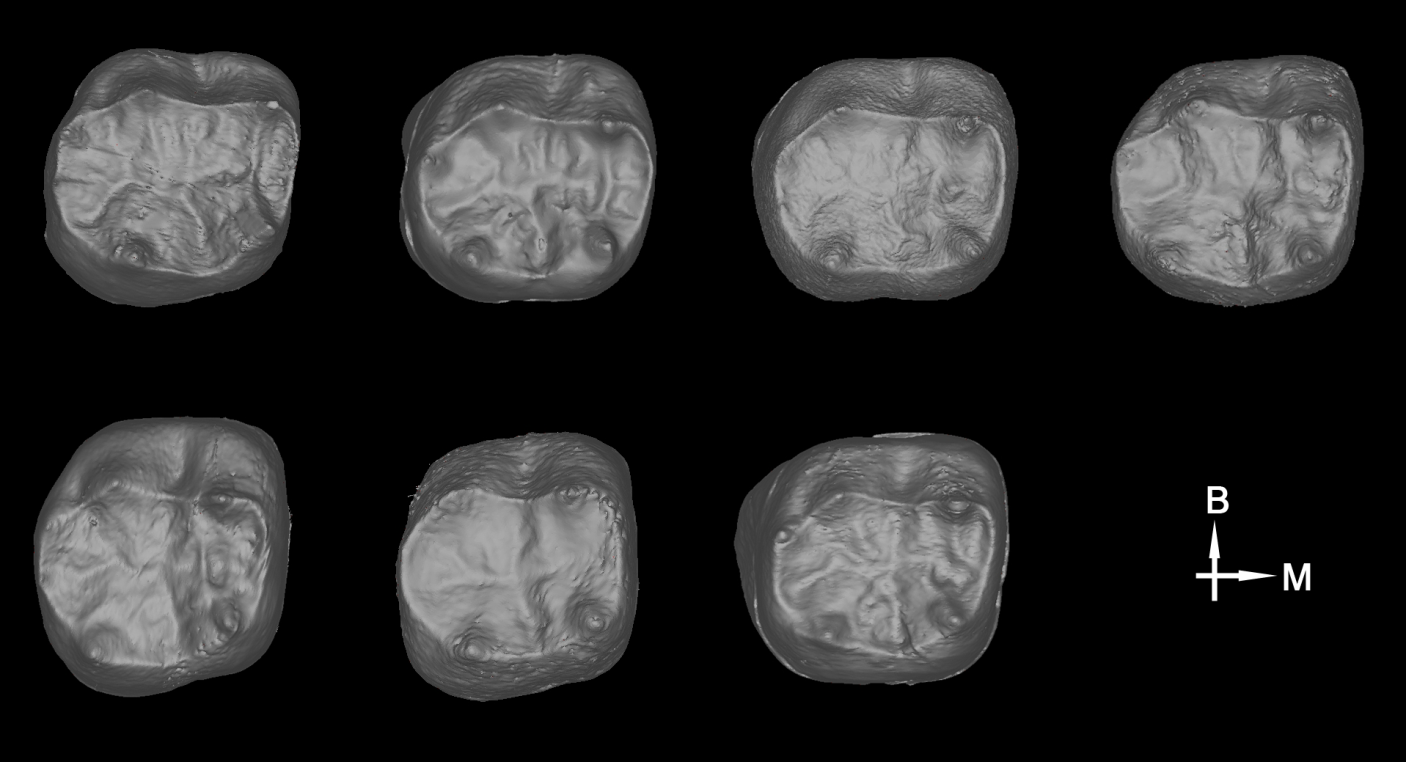


SI Figure 10. The EDJ surfaces of recent modern human M_1_s. B: buccal, M: mesial.


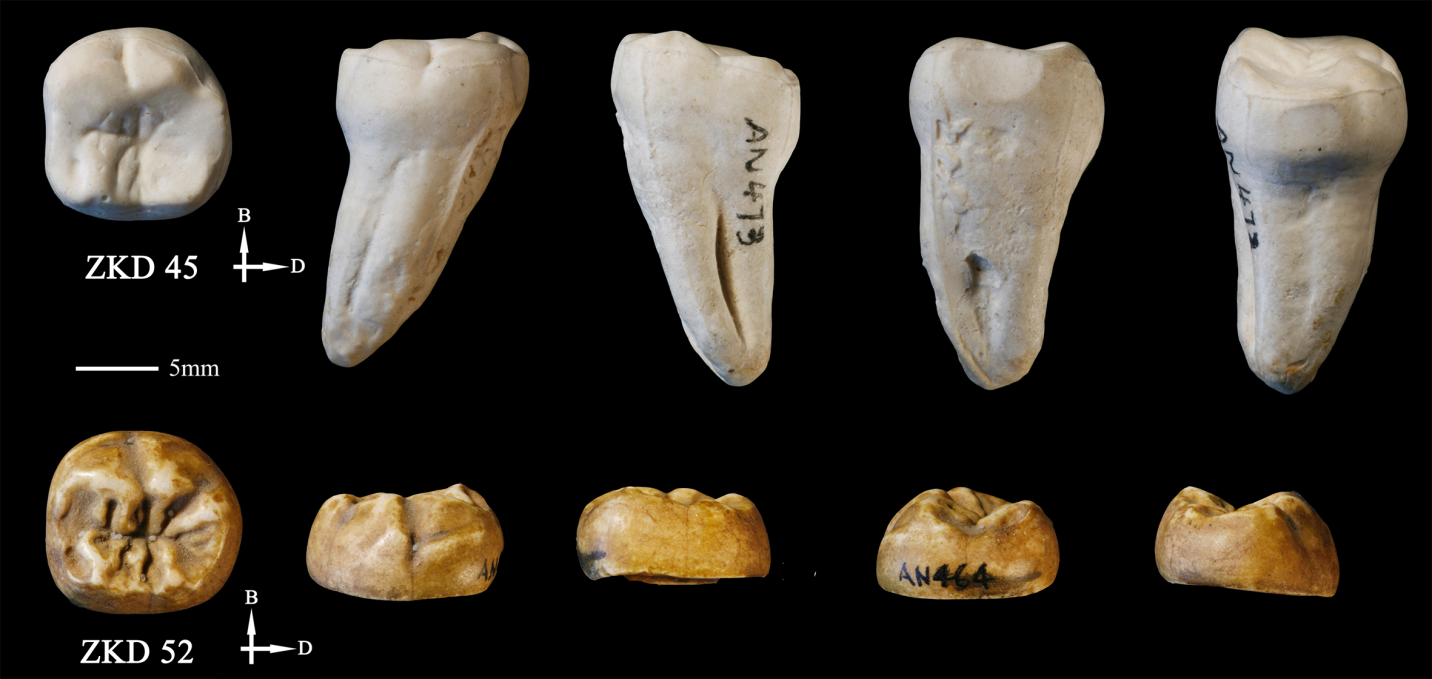


SI Figure 11. Lower second molars of Zhoukoudian. From left to right are occlusal, buccal, lingual, mesial, and distal views. B: buccal, D: distal.


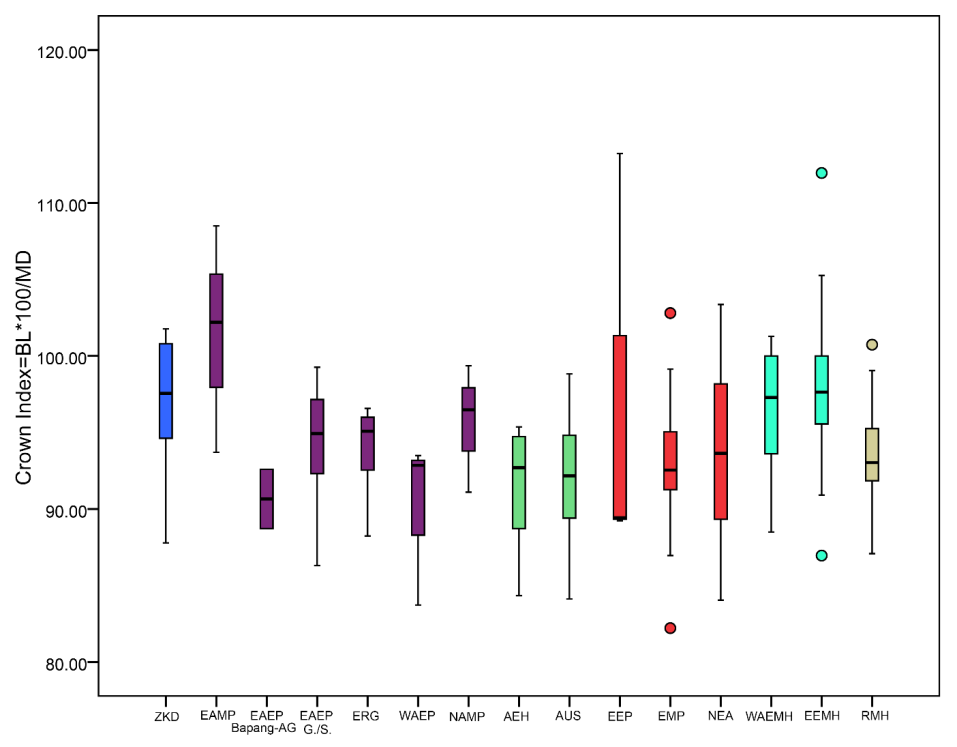


SI Figure 12. Boxplots of crown indices of Zhoukoudian M_2_s and other comparative specimens. ZKD: Zhoukoudian; EAMP: East Asian Middle Pleistocene; EAEP Bapang-AG: East Asian Early Pleistocene-Sangiran Bapang-AG assemblage; EAEP G./S.: East Asian Early Pleistocene-Sangiran Grenzbank/Sangiran assemblage; ERG: *Homo* *ergaster*; WAEP: West Asian Early Pleistocene; NAMP: North African Middle Pleistocene; AEH: African early *Homo*; AUS: *Australopithecus*; EEP: European Early Pleistocene; EMP: European Middle Pleistocene; NEA: Neanderthals; WAEMH: West Asian early modern human; EEMH: European early modern human; RMH: Recent modern human.


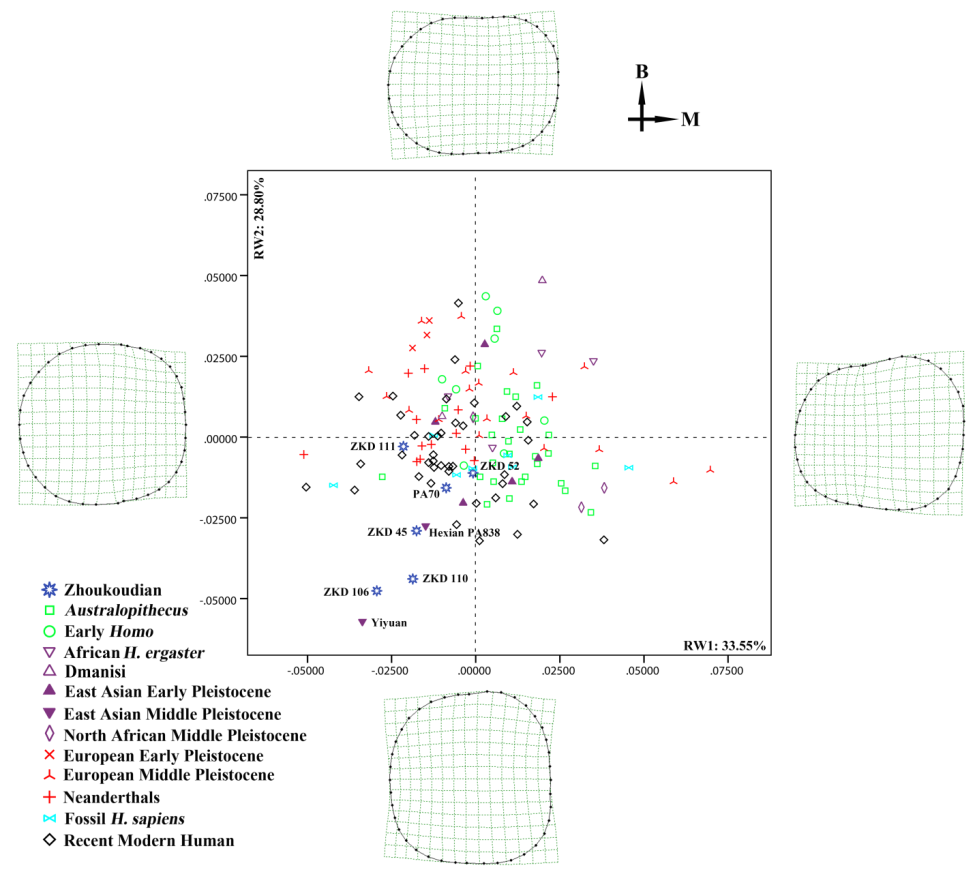


SI Figure 13. Geometric morphometric analyses of M_2_ crown outline shapes (TPS-grids correspond to the positive-value or negative-value extremes of the RW axis). B: buccal; M: mesial.


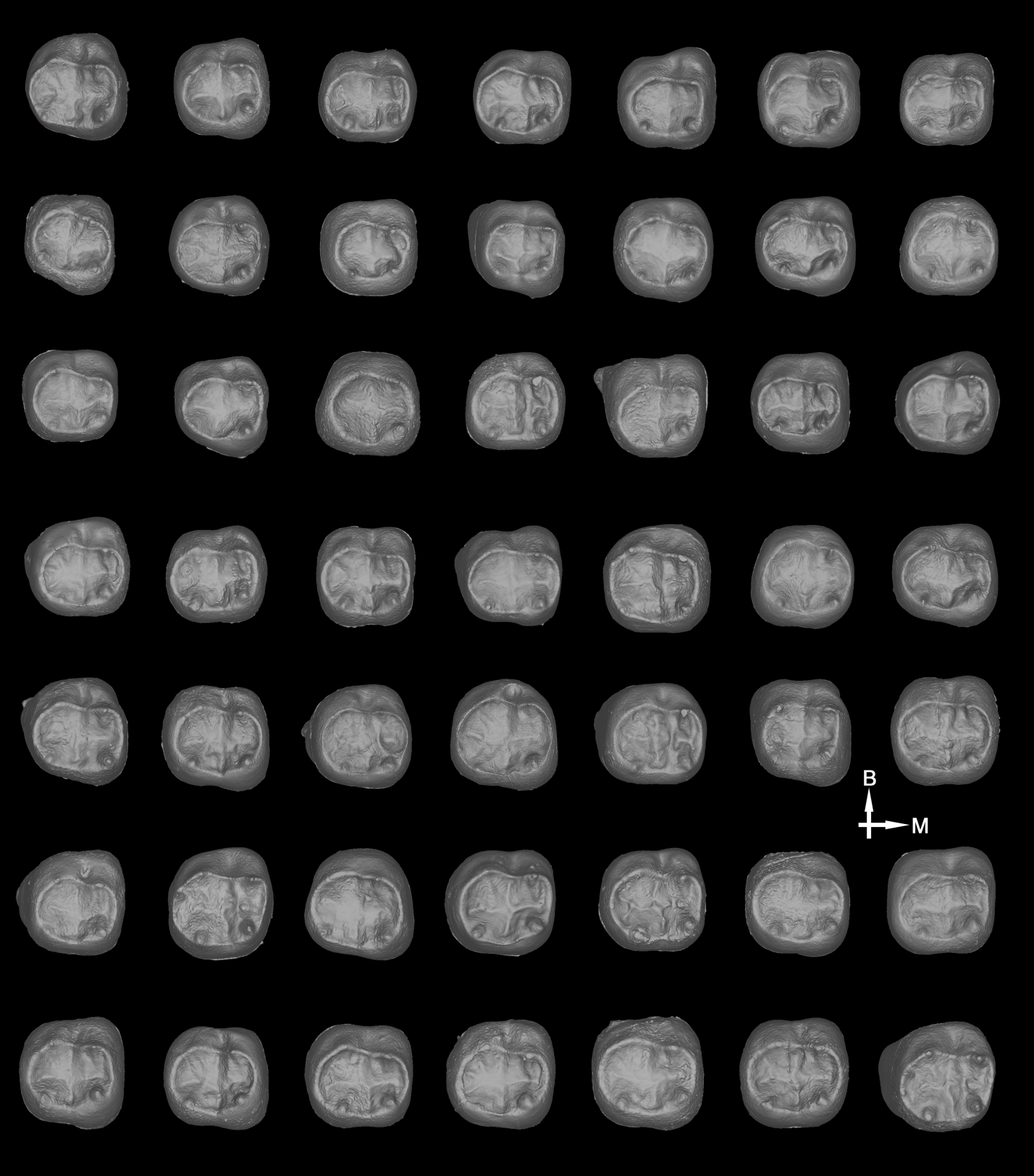


SI Figure 14. The EDJ surfaces of recent modern human M_2_s. B: buccal, M: mesial.


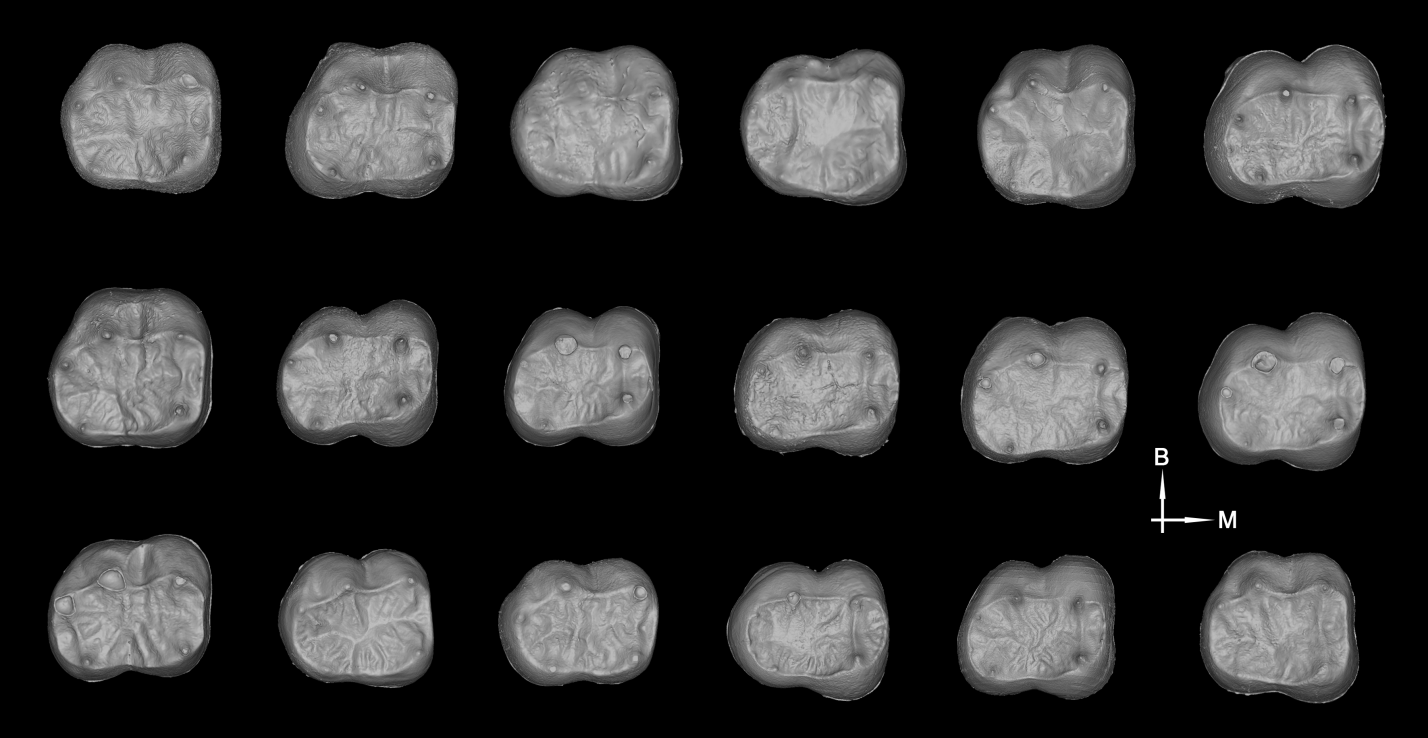


SI Figure 15. The EDJ surfaces of *Pongo* M_2_s. B: buccal, M: mesial.


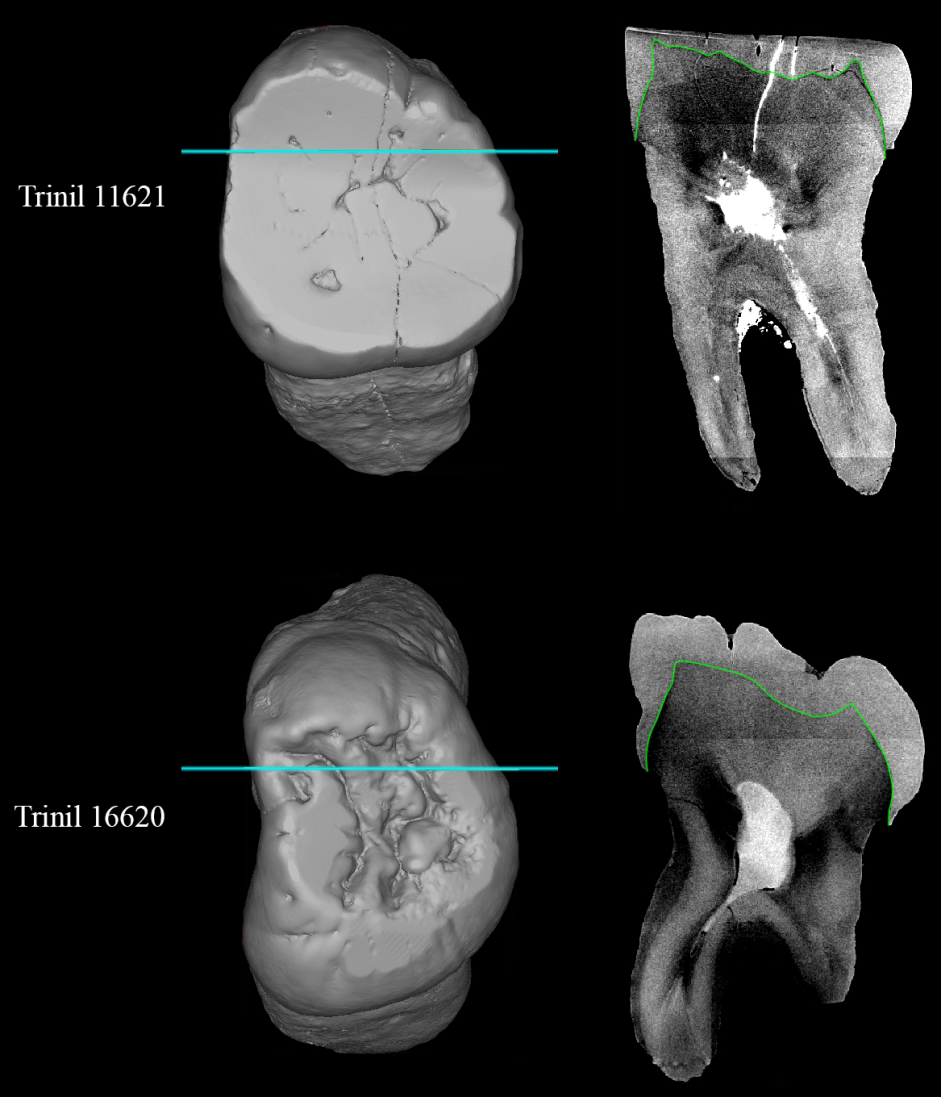


SI Figure 16. The sagittal section of two M^3^s from Trinil, Indonesian (The CT data is from European Synchrotron Radiation Facility [ESRF] [http://paleo.esrf.fr] and published in Smith et al^38^).

SI Table 1. Morphological comparisons of the Zhoukoudian hominins and other members of *H. erectus* sensu lato.

|  |  | Zhoukoudian | *H. ergaster* | East Asian Early Pleistocene | East Asian mid-Middle Pleistocene |
| --- | --- | --- | --- | --- | --- |
| I^1^ | Shovel shape | Pronounced | Moderate | Faint to moderate | Pronounced |
|  | Labial convexity | Moderate | Weak to Moderate | Faint to strong | Moderate |
|  | Numbers of finger-like projections | 2-5 | 2-5 | 0 | 5 |
|  | Lingual central ridge | 3/4 | 1/ 2 | 2/3 | 0/1 |
|  | Buccal surface | Highly wrinkled | Relatively smooth | Relatively smooth | Highly wrinkled |
| P^3^ | Transverse crest | 1/ 3 | 1/ 4 | 4/ 9 | 2/ 5 |
|  | Root number | 2 (coalesced or not) | 2 (coalesced or not) to 3 | 2 to 3 | 2 (coalesced or not) to 3 |
| P^4^ | Crown outline shape | Lingual part is no wider than the buccal part | Lingual part can be wider than the buccal part | Lingual part is no wider than the buccal part | Lingual part is no wider than the buccal part |
|  | Transverse crest | 2/4 | 4/ 4 | 4/ 7 | 0/2 |
|  | Root number | 1 or 2 | 2 (coalesced or not) | 2 | 2 (coalesced) |
| P_3_ | Talonid | Presence/Absence | Presence | Presence | Presence |
|  | Buccal vertical groove | Absent to Pronounced | Absent to Strong | Faint to Strong | --- |
|  | Root | 1R to 1T (Tomes’ root with bifid tip) | 1T to 2R: MB+D | 1T to 2R: MB+D | --- |
| M_1_ | EDJ surface | Highly crenulated | --- | --- | Relatively simple |
|  | Middle trigonid crest | 1/7 | 3/ 8 | 4/11 | 1/ 1 |
|  | Protostylid | Pit-like to Pronounced | Absent to Pronounced | Absent to Pronounced | Moderate |
| M_2_ | Crown outline shape | BL-expanded rounded | MD-elongated elliptical | MD-elongated elliptical or BL-expanded rounded | BL-expanded rounded |
|  | EDJ surface | Highly crenulated | --- | --- | Highly crenulated/Relatively simple |
|  | Middle trigonid crest | 0/7 | 3/7 | 1/13 | 1/5 |
|  | Root structure | Coalesced to Highly bifurcated | Highly bifurcated | Highly bifurcated | Coalesced to Highly bifurcated |
|  | Taurodontism | Yes | --- | --- | No |

*The degrees of buccal vertical groove is scored gradiently as follows: 1) Smooth; 2) Faint or Depression; 3) Weakly developed; 4) Moderate; 5) Pronounced; 6) Strong, and slightly rolled.

* The degrees of the P_3_ root is scored following system in Wood et al. (1988)^27^

SI Table 2. The independent t-test of crown index (BL*100/MD) between Zhoukoudian M_1_s and other taxonomic groups.

|  | EAEP Bapang-AG (n=3) | EAEP G./S. (n=9) | ERG (n=5) | WAEP (n=3) | NAMP (n=3) | AEH (n=9) | AUS (n=26) | EEP (n=2) | EMP (n=23) | NEA (n=53) | WAEMH (n=11) | EEMH (n=30) | RMH (n=42) |
| --- | --- | --- | --- | --- | --- | --- | --- | --- | --- | --- | --- | --- | --- |
| ZKD (n=12) | .104 | .864 | **.010**** | .093 | .814 | **.018*** | .088 | .067 | .959 | .579 | .197 | .255 | .816 |

* ZKD: Zhoukoudian; EAEP Bapang-AG: East Asian Early Pleistocene-Sangiran Bapang-AG assemblage; EAEP G./S.: East Asian Early Pleistocene-Sangiran Grenzbank/Sangiran assemblage; ERG: *Homo* *ergaster*; WAEP: West Asian Early Pleistocene; NAMP: North African Middle Pleistocene; AEH: African early *Homo*; AUS: *Australopithecus*; EEP: European Early Pleistocene; EMP: European Middle Pleistocene; NEA: Neanderthals; WAEMH: West Asian early modern human; EEMH: European early modern human; RMH: Recent modern human.

SI Table 3. The independent t-test of crown index (BL*100/MD) between Zhoukoudian M_2_s and other taxonomic groups.

|  | EAMP (n=3) | EAEP Bapang-AG (n=2) | EAEP G./S. (n=7) | ERG (n=5) | WAEP (n=3) | NAMP (n=3) | AEH (n=9) | AUS (n=27) | EEP (n=3) | EMP (n=29) | NEA (n=42) | WAEMH (n=8) | EEMH (n=26) | RMH (n=51) |
| --- | --- | --- | --- | --- | --- | --- | --- | --- | --- | --- | --- | --- | --- | --- |
| ZKD (n=8) | .251 | .119 | .270 | .213 | .068 | .690 | **.017*** | **.003**** | .969 | **.023*** | .138 | .834 | .593 | **.005**** |

* ZKD: Zhoukoudian; EAMP: East Asian Middle Pleistocene; EAEP Bapang-AG: East Asian Early Pleistocene-Sangiran Bapang-AG assemblage; EAEP G./S.: East Asian Early Pleistocene-Sangiran Grenzbank/Sangiran assemblage; ERG: *Homo* *ergaster*; WAEP: West Asian Early Pleistocene; NAMP: North African Middle Pleistocene; AEH: African early *Homo*; AUS: *Australopithecus*; EEP: European Early Pleistocene; EMP: European Middle Pleistocene; NEA: Neanderthals; WAEMH: West Asian early modern human; EEMH: European early modern human; RMH: Recent modern human.

SI Table 4. Fossil hominin teeth from the Zhoukoudian Locality 1^a^.

| Specimen No. | Tooth category | Layers | Individual | Crown measurements (mm)^b^ | | Occlusal wear score |
| --- | --- | --- | --- | --- | --- | --- |
|  |  |  |  | MD | BL |  |
| PA66 | Left upper central incisor (I^1^) | 8-9 | I | 10.7 | 8.1 | 3 |
| PA67 | Right upper third premolar (P^3^) | 11 | II | 8.7 | 11.9 | 3 |
| PA68 | Right upper forth premolar (P^4^) | 11 | II | 8.2 | 11.7 | 3 |
| PA69 | Left lower third molar (M_1_) | 3 | III | 12.7 | (12.0) | 3 |
| PA70 | Left lower forth molar (M_2_) | 8-9 | IV | 12.3 | 11.8 | 2 |
| PA110 | Right lower third premolar (P_3_) | 8-9 | V | 7.9 | 8.2 | 3 |

^a^Crown measurements in parentheses are values estimated by Wu and Chia (1954)^39^ due to the incompleteness of crown (see Figure 3). MD: mesiodistal dimension; BL: buccolingual dimension. ^b^crown measurements were cited from Wu and Chia (1954)^39^ and Qiu et al. (1973)^40^.

SI Table 5. The six original teeth of Peking Man and other teeth of the same categories were aligned with different natural layers from which they were recovered. The dating results attained through various methods were provided for each natural layer. TL: thermoluminescence; ESR: electron spin resonance; TIMS: Thermal ionization mass spectrometric.

| Layers | Dating methods and results (kyr) | | | | | | Dental categories and specimens | | | | | |
| --- | --- | --- | --- | --- | --- | --- | --- | --- | --- | --- | --- | --- |
|  | Fission Track^1^ | U-series^2,3^ | TL^4^ | ESR^5^ | TIMS^6^ | ^26^Al/^10^Be^7^ | I^1^ | P^3^ | P^4^ | P_3_ | M_1_ | M_2_ |
| 3 |  | 230±50 |  | 282 | 400-500 |  |  |  |  | **PA 110** |  |  |
| 4 | 299±55 | 30±40 | 292±26~312±28 |  |  |  | ZKD 1, 2 |  |  | ZKD 80, 81 | ZKD 43, 97, 98 |  |
| 5 |  |  |  |  | 486-640 |  |  |  | ZKD 25 | ZKD Z | ZKD 34, | ZKD 106 |
| 6 |  |  |  |  |  |  |  |  |  |  |  |  |
| 7 |  |  |  |  |  |  |  |  |  | ZKD 82 | ZKD 100 | ZKD 110 |
| 8-9 |  | >35 | 423±80 | 418 |  | 670±290~750±160 | **PA66**  ZKD 3 | ZKD 77, 78 | ZKD 28, 87, 88, 133’ | ZKD 20, 21, 85, 130’ | **PA 69**  ZKD 36, 38, 102, 137’ | **PA 70**  ZKD 45, 107, 111 |
| 10 | 452±44 |  | >417 <592 |  |  | 750±210 |  |  |  |  |  |  |
| 11 |  |  | 585±105 | 578 |  |  | ZKD 4 | **PA67**  ZKD 19 | **PA68**  ZKD 27 |  | ZKD 99 | ZKD 52, 108 |

*1: Guo et al., 1991^41^; 2: Zhao et al., 1985^42^; 3: Yuan et al., 1991^43^; 4: Pei, 1985^44^; 5: Huang et al., 1991^45^; 6: Shen et al., 2001^46^; 7: Shen et al., 2009^47^

* In Weidenreich (1937)^14^, Zhoukoudian teeth were cataloged as *Sinanthropus*+number. Since the *Sinanthropus* has been lumped into the group of *H. erectus*, this study will use a more concise cataloguing way, ZKD+number. ZKD means Zhoukoudian, and the numbers will stay the same as Weidenreich’s. The specimens (1 M^3^, 1P_3_, and 1 P_4_) published by Zdansky (1926, 1952)^48,49^ will be referred as ZKD Z.

* The six fossil teeth of Peking Man were marked in bold.

SI Table 6. Specimens used in the morphological comparisons.

| Geography and Chronology | Specimens |
| --- | --- |
| **Africa** |  |
| Pliocene (*Australopithecus*) (n=120) | MLD2, 5, 11, 23, 43, 45; Stw1, 3, 7, 14, 16, 18, 19, 35, 45, 50, 54, 60, 61, 67, 72, 73, 75l, 91, 97, 104, 106, 107, 109, 120, 123, 130, 131, 133, 134,138, 141, 142, 145, 148, 149, 151, 183a, 192a, 193, 195, 202, 203, 213, 231, 233, 234, 235, 240, 246, 252, 269, 276, 280, 285, 286, 291, 306, 308, 309, 321, 327, 364, 384, 386, 401, 404, 408, 409, 412, 417, 419, 420, 421, 424, 427, 429, 430, 480, 492, 498, 519, 534, 540, 541, 555, 560, 566, 574, 750 |
| Late Pliocene and Early Pleistocene (Early *Homo*) (n=61) | KNM ER-808, 809, 1470, 1482, 1483A, 1502, 1506, 1590, 1802, 1805, 1813, 1814, 2597, 3891, 5431; OH6, 7, 13, 16, 24, 37, 39; Omo 29-43(1968), Omo75s-15, Omo123-5495, Omo177-4525(1973), Omo195-1630, K7-19 (1969), L26-1g, L28-31, L7-279, L628-10, L824-5, L894-1 |
| Late Pliocene and Early Pleistocene (*H. ergaster*) (n=30)  North African Middle Pleistocene (n=9) | KNM ER-730, 731, 803, 806, 820, 992, 1507, 1808, 1811, 1812, 3733; KNM WT-15000; Stw80  Ternifine 1, 2, 3 |
| **East Asia** |  |
| Early Pleistocene (n=56) | BK7905; Ng8503; SB8103; S1b, 4, 5, 6, 7-1, 7-3a, 7-20, 7-25, 7-26, 7-27, 7-29, 7-30, 7-31, 7-32, 7-34, 7-35, 7-36, 7-42, 7-43, 7-48, 7-58, 7-61, 7-62, 7-63, 7-64, 7-65, 7-76, 7-78, 7-84, 7-85, 7-86, 7-49, 9, 15, 16, 17, 22, 27; Trinil 5; Yuanmou |
| mid-Middle Pleistocene (n=13)  Late Middle Pleistocene (n=5) | Hexian HXUP3, PA831, PA832, PA834-1, PA835, PA838, PA839; Hualong Cave; Xichuan PA524, PA533; Yiyuan Sh.y.003, Sh.y.007, Sh.y.072  Chaoxian; Panxian Dadong (PDH1, PDH3, PDH4) |
| Holocene (Recent modern human) (n=463) | Henan and Hubei Province |
| **West Asia** |  |
| Early Pleistocene (n=16) | Dmanisi (D211, D2282, D2600, D2700, D2735, D2736, D3672) |
| Late Pleistocene (Early modern human) (n=15) | Qafzeh 4, 5, 6, 7, 9, 11 |
| **Europe** |  |
| Early Pleistocene (n=13) | Atapuerca Gran Dolina (ATD6-3, ATD6-5, ATD6-7, ATD6-8, ATD6-9, ATD6-13, ATD6-69, ATD6-96, ATD6-113, ATD6-144) |
| Middle Pleistocene (n=5) | Arago (6, 16, 26, 32, 36) |
| Neanderthals (n=14) | Arcy Grotte Renne (5, 8, 13, 21, 35, 41); Arcy Hyene (4); Krapina (49, Md C, Md D, Md E, Md G57, Md H, Md J); Regourdou 1; La Quina (H5); St. Césaire 1; Hortus (II, IV); Monsempron; Petit-Puymoyen |
| Late Pleistocene (Early modern human) (n=21) | Abri Pataud 1; Dolní Vĕstonice (13, 14); Les Rois; Saint Germain-La Riviere (SG-LRB4, SG-LRB5, SG-LR6) |

SI Table 7. Specimens used in the linear metric comparisons.

| Geography and Chronology | Specimens | Reference |
| --- | --- | --- |
| **Africa** |  |  |
| Pliocene (*Australopithecus*) (n=53) | Stw1, 3, 14, 61, 67, 72, 97, 104, 106, 109, 120, 123, 130, 131, 133, 134, 141, 149, 151, 213, 234, 235, 246, 269, 286, 291, 308, 309, 321, 327, 364, 384, 386, 404, 409, 412, 424, 429, 492, 498c, 519, 534, 540, 541, 555, 560d, 566 | [8] |
| Late Pliocene and Early Pleistocene (Early *Homo*) (n=18) | KNM ER-1482, 1502, 1506, 1802, 1805, 1814, 3734; OH7, 13, 16, 37 | [1] |
| Late Pliocene and Early Pleistocene (*H. ergaster*) (n=10) | KNM ER-730, 806, 820, 992, 1507, 1808; KNM WT-15000 | [1, 10] |
| North African Middle Pleistocene (n=6) | Ternifine 1, 2, 3 | [1] |
| **East Asia** |  |  |
| Early Pleistocene (n=21) | BK7905, Ng8503, SB8103, Sangiran 1b, 6a, 7-20, 7-42, 7-43, 7-61, 7-62, 7-64, 7-65, 7-76, 7-78, 7-84, 9, 22 | [4, 5, 9] |
| mid-Middle Pleistocene (n=22) | Hexian PA838, 839; Yiyuan Sh.y.072; Zhoukoudian ZKD34, 35, 36, 38, 43, 44, 45, 52, 96, 98, 99, 100, 102, 106, 110, 111, 137', 138’, 147’ | [11, 12, 13] |
| **West Asia** |  |  |
| Early Pleistocene (n=6) | Dmanisi (D211, D2375, D2600, D2735) | [6] |
| Late Pleistocene (Early modern human) (n=19) | Qafzeh 3, 4, 7, 8, 9, 10, 11, 12, Q79-C11; Skhul2, 4, 5 | [1] |
| **Europe** |  |  |
| Early Pleistocene (n=5) | Atapuerca Gran Dolina (ATD6-5, ATD 6-69, ATD 6-96, ATD 6-113) | [2, 3] |
| Middle Pleistocene (n=52) | Arago (1, 2, 13); Atapuerca Sima de los Huesos (AT-1-I, AT-2-II, AT-11-XIII, AT-14-IV, AT-21/1957-VII, AT-22/101-III, AT-75-VI, AT-1759-VI, AT-141/556-X, AT-169-X,AT-272/286-XI, AT-273/271-III, AT-285/2779-XVI, AT-300/4147-XII, AT-505-XIX, AT-557/1761-XI, AT-561/1775-XXVI, AT-576-XIX, AT-605/605-XXII, AT-607/607-XXIII, AT-792/792-XXVII, AT-793-IV, AT-829/943-XVIII, AT-888/888-XXI, AT-941/1752-XVIII, AT-946/3890-XX, AT-950/950-XXVIII AT-1458/2438-XXIV, AT-1459/2276-XIV, AT-1957-VII, AT-2193-XV, AT-2193-XV, AT-2270/1756-XXVI, AT-2272/284-XIV, AT-2396/2438-XXIV, AT-2763-XVI, AT-3175/4318-XX, AT-3176/792-XXVII, AT-3179/421-II, AT-3889-XXV, AT-3933/3934-XXV, AT-4147/300-XII; Mauer; Montmaurin | [1, 7] |
| Neanderthals (n=95) | Arcy-sur-Cure (8, H1, L1=#5); Amud (1, 5); Chateauneuf 2; Ehringsdorf (6, 7, 8); Genay (Côte d'Or) 1; Hortus (4, 5, 1262); Krapina (6, 80, 86, 104, 105, 107, A/B, D/D, F/H, N/N, Md C, Md E, Md G, Md J, Md L, Md M, Md P, Md Q); La Chaise (9, 13, 14); La Quina (5, 9); Le Moustier 1; Ochoz 1; Petit Puymoyen (1, 2, 3); Regourdou 1; Spy (1, 2); Sakajia 2; Shanidar (1, 2, 4, 6); St. Césaire 1; Subalyuk 1; Tabun (1, 2, 3-1, 3-3, 3-4, EB); Vindija (206, 226, 231) | [1] |
| Late Pleistocene (Early modern human) (n=56) | Abri Pataud (1, 26.244); Brno (Zabovresky) 3; Combe Capelle 1; Dolní Vĕstonice 3; Isturits (4, 7B-UNN); Le Rois (R48, R50-3, R5-10, R50-31, R50-40); Les Vachons 1; Mladeč (1903 Mnd 1, 1904 Mnd5); Pavlov 1; Predmostí (1, 2, 3, 4, 5, 7, 9, 10, 14, 18, 25, 26, 27 ,259, 476, 3070) | [1] |
| Holocene (Recent modern human) (n=93) | Henan and Hubei Province (LM1=42; LM2=51) | [0] |

^*^0: Present study; 1: Wolpoff (personal communication); 2: Bermúdez de Castro et al., 1999^50^; 3: Carbonell et al., 2005^51^; 4: Grine and Franzen, 1994^6^; 5: Kaifu et al., 2005^24^; 6: Martinón-Torres et al., 2008^7^; 7: Martinón-Torres et al., 2012^8^; 8: Moggi-Cecchi et al., 2006^13^; 9: Tobias and von Koenigswald, 1965^52^; 10: Walker and Leakey, 1993^12^; 11: Weidenreich, 1937^14^; 12: Xing et al., 2015^10^; 13: Xing et al., 2016^15^

SI Table 8. Specimens used in the geometric morphometric analyses.

| Geography and Chronology | Specimens |
| --- | --- |
| **Africa** |  |
| Pliocene (*Australopithecus*) (n=40) | AL128-23, 145-35, 188-1, 207-13, 266-1, 277-1, 288-1, 333-12, 333w-60; LH3, 4; MLD2; Stw14, 109, 120, 142, 195, 213, 233, 234, 235, 269, 286, 308, 384, 404, 412, 424, 427, 498c, 498d, 519, 534, 540, 555, 560d |
| Late Pliocene and Early Pleistocene (Early *Homo*) (n=17) | KNM ER-1506, 1802, 1814, 3734; OH7, 13, 16; Omo 29-43-1968, Omo123-5495, Omo177-(73)-4525, L7-279, L628-10 |
| Late Pliocene and Early Pleistocene (*H. ergaster*) (n=8)  North African Middle Pleistocene (n=6) | KNM ER-806, 992; KNM WT-15000; Stw80  Ternifine 1, 2, 3 |
| Holocene (Recent modern human) (n=40) | South Africa (LM1=20; LM2=20) |
| **East Asia** |  |
| Early Pleistocene (n=11) | S1b, 6, 7-25, 7-26, 7-64, 7-65, 7-69, 9; Trinil 5 |
| mid-Middle Pleistocene (n=2) | Hexian PA838; Yiyuan Sh.y.072 |
| Holocene (Recent modern human) (n=40) | Henan and Hubei Province (LM1=20; LM2=20) |
| **West Asia** |  |
| Early Pleistocene (n=4)  Late Pleistocene (Early modern human) (n=1) | Dmanisi (D211, D2375)  Qafzeh 7 |
| **Europe** |  |
| Early Pleistocene (n=5) | Atapuerca Gran Dolina (ATD6-3, ATD6-5, ATD6-96, ATD6-113) |
| Middle Pleistocene (n=31) | Arago (10, 13, 32); Atapuerca Sima de los Huesos (AT-1, 148, 169, 273, 300, 563, 607, 807, 941, 1466, 1761, 1993, 2193, 2396, 2438, 2763, 2767, 3045, 3176, 3179, 3243, 3889, 3890, 3941, 4100, 4328); Montmarin |
| Neanderthals (n=19) | Arcy Grotte Tenne (5, 21); Arcy Hyene 4; Hortus 4; Krapina D, E, G57, H, J; Regourdou 1; St. Césaire 1; Subalyuk 1; Vindija 206, 231 |
| Late Pleistocene (Early modern human) (n=7) | Abri Pataud 1; Dolní Vĕstonice 13, 14; Le Rois; Saint Germain-La Riviere (SG-LR6) |

SI Table 9. The background of Xichuan and Hualong Cave hominins.

|  | Location | Taxon | Chronology | Dating methods |
| --- | --- | --- | --- | --- |
| Xichuan^a^ | Xichuan, Henan | *H. erectus* | Unknown | --- |
| Hualong Cave^b^ | Dongzhi, Anhui | *H. erectus* | Middle Pleistocene | Biostratigraphy |

a See Wu and Wu, 1982^53^.

b See Gong et al., 2014^54^.

**References**

1 Molnar, S. Human tooth wear, tooth function and cultural variability. *American Journal of Physical Anthropology* **34**, 175-189 (1971).

2 Hlusko, L. J. Protostylid variation in *Australopithecus*. *Journal of Human Evolution* **46**, 579-594 (2004).

3 Scott, G. R. & Turner, C. G. *The anthropology of modern human teeth: dental morphology and its variation in recent human populations*. Vol. 20 (Cambridge University Press, 1997).

4 Martinón-Torres, M. *et al.* Dental evidence on the hominin dispersals during the Pleistocene. *Proceedings of the National Academy of Sciences of the United States of America* **104**, 13279-13282 (2007).

5 Wood, B. *Hominid cranial remains. Koobi Fora research project, Vol. 4*. (Clarendon Press, 1991).

6 Grine, F. & Franzen, J. Fossil hominid teeth from the Sangiran dome (Java, Indonesia). *Courier Forschungs-Institut Senckenberg* **171**, 75-103 (1994).

7 Martinón-Torres, M. *et al.* Dental remains from Dmanisi (Republic of Georgia): Morphological analysis and comparative study. *Journal of Human Evolution* **55**, 249-273 (2008).

8 Martinón-Torres, M., Bermúdez de Castro, J. M., Gómez-Robles, A., Prado-Simón, L. & Arsuaga, J. L. Morphological description and comparison of the dental remains from Atapuerca-Sima de los Huesos site (Spain). *Journal of Human Evolution* **62**, 7-58 (2012).

9 Xing, S., Martinón-Torres, M., Bermúdez de Castro, J. M., Wu, X. & Liu, W. Hominin teeth from the early Late Pleistocene site of Xujiayao, Northern China. *American Journal of Physical Anthropology* **156**, 224-240 (2015).

10 Xing, S. *et al.* Middle Pleistocene Hominin Teeth from Longtan Cave, Hexian, China. *PloS ONE* **9**, e114265 (2015).

11 Liu, W. *et al.* Late Middle Pleistocene hominin teeth from Panxian Dadong, South China. *Journal of Human Evolution* **64**, 337-355 (2013).

12 Walker, A. & Leakey, R. E. *The Nariokotome Homo erectus skeleton*. (Harvard University Press, 1993).

13 Moggi-Cecchi, J., Grine, F. E. & Tobias, P. V. Early hominid dental remains from Members 4 and 5 of the Sterkfontein Formation (1966–1996 excavations): Catalogue, individual associations, morphological descriptions and initial metrical analysis. *Journal of Human Evolution* **50**, 239-328 (2006).

14 Weidenreich, F. *The dentition of Sinanthropus pekinensis: a comparative odontography of the hominids*. Palaeontologica Sinica, New Series D 1, 1-180 (1937).

15 Xing, S. *et al.* Hominin teeth from the Middle Pleistocene site of Yiyuan, Eastern China. *Journal of Human Evolution* **95**, 33-54 (2016).

16 Tobias, P. V. *Olduvai Gorge (The skulls, endocasts and teeth of Homo habilis, Vol. 4)*. (Cambridge University Press, 1991).

17 Kaifu, Y. Advanced dental reduction in Javanese *Homo erectus*. *Anthropological Science* **114**, 35-43 (2006).

18 Shaw, J. C. Taurodont Teeth in South African Races. *Journal of Anatomy* **62**, 476-498.471 (1928).

19 Kupczik, K. & Hublin, J.-J. Mandibular molar root morphology in Neanderthals and Late Pleistocene and recent *Homo sapiens*. *Journal of Human Evolution* **59**, 525-541 (2010).

20 Kallay, J. Some anomalies of the lower premolar roots of *Homo primigenius* from Krapina in Croatia. *Folia Stomatol (Zagreb)* **10**, 25-36 (1949).

21 Kallay, J. A radiographic study of the Neanderthal teeth from Krapina, Croatia. In (D. R. Brothwell, Ed.) *Dental Anthropology*, pp.75–86. (Pergamon Press, 1963).

22 Madeira, M. C., Leite, H. F., Niccoli Filho, W. D. & Simões, S. Prevalence of taurodontism in premolars. *Oral Surgery Oral Medicine Oral Pathology* **61**, 158 (1986).

23 Llamas, R. & Jimenezplanas, A. Taurodontism in premolars. *Oral Surgery Oral Medicine & Oral Pathology* **75**, 501-505 (1993).

24 Kaifu, Y., Aziz, F. & Baba, H. Hominid Mandibular Remains from Sangiran: 1952–1986 Collection. *American Journal of Physical Anthropology* **128**, 497-519 (2005).

25 Zanolli, C. & Mazurier, A. Endostructural characterization of the *H. heidelbergensis* dental remains from the early Middle Pleistocene site of Tighenif, Algeria. *Comptes Rendus Palevol* **12**, 293-304 (2013).

26 Bräuer, G. & Schultz, M. The morphological affinities of the Plio-Pleistocene mandible from Dmanisi, Georgia. *Journal of Human Evolution* **30**, 445-481 (1996).

27 Wood, B., Abbott, S. & Uytterschaut, H. Analysis of the dental morphology of Plio-Pleistocene hominids. IV. Mandibular postcanine root morphology. *Journal of Anatomy* **156**, 107-139 (1988).

28 Leakey, L. S., Tobias, P. V. & Napier, J. R. A new species of the genus *Homo* from Olduvai Gorge. *Nature* **202**, 7 (1964).

29 Martínez de Pinillos, M. *et al.* Trigonid crests expression in Atapuerca-Sima de los Huesos lower molars: Internal and external morphological expression and evolutionary inferences. *Comptes Rendus Palevol* **13**, 205-221 (2014).

30 Bailey, S. E. A closer look at Neanderthal postcanine dental morphology: The mandibular dentition. *The Anatomical Record* **269**, 148-156 (2002).

31 Skinner, M. M., Gunz, P., Wood, B. A. & Hublin, J.-J. Enamel-dentine junction (EDJ) morphology distinguishes the lower molars of *Australopithecus africanus* and *Paranthropus robustus*. *Journal of Human Evolution* **55**, 979-988 (2008).

32 Zanolli, C. *et al.* The late Early Pleistocene human dental remains from Uadi Aalad and Mulhuli-Amo (Buia), Eritrean Danakil: macromorphology and microstructure. *Journal of Human Evolution* **74**, 96 (2014).

33 Bailey, S. E., Skinner, M. M. & Hublin, J.-J. What lies beneath? An evaluation of lower molar trigonid crest patterns based on both dentine and enamel expression. *American Journal of Physical Anthropology* **145**, 505-518 (2011).

34 Martinón-Torres, M. *et al.* Talonid crests expression at the enamel–dentine junction of hominin lower permanent and deciduous molars. *Comptes Rendus Palevol* **13**, 223-234 (2014).

35 Zanolli, C. Molar crown inner structural organization in Javanese *Homo erectus*. *American Journal of Physical Anthropology* **156**, 148-157 (2015).

36 Blumberg, J. E., Hylander, W. L. & Goepp, R. A. Taurodontism: A biometric study. *American Journal of Physical Anthropology* **34**, 243-255 (1971).

37 Liu, W. *et al.* A mandible from the Middle Pleistocene Hexian site and its significance in relation to the variability of Asian *Homo erectus*. *American Journal of Physical Anthropology* **162**, 715-731 (2017).

38 Smith et al. Taxonomic assessment of the Trinil molars-Using non-destructive 3D structural and development analysis. *PaleoAnthropology* **2009**, 117-129 (2009)

39 Wu, J.-K. & Chia, L.-P. New discovery of a *Sinanthropus pekinensis* in Choukoutien. *Acta Palaeontologica Sinica* **2**, 267-288 (1954).

40 Qiu, Z., Gu, Y., Zhang, Y. & Zhang, S. Newly discovered Sinanthropus remains and stone artifacts at Zhoukoudian. *Vertebrata Palasiatica* **11**, 109-131 (1973).

41 Guo, S. L. *et al*. Fission track dating of 4th Layer of the Peking Man site. *Acta Anthropologica Sinica* **10,** 73–77, (1991).

42 Zhao, S. S. *et al*. Study of chronology of Peking Man site. In (R. K. Wu, M. E. Ren, X. M. Zhu, Z. G. Yang, C. K. Hu, Z. C. Kong, Y. Y. Xie & S. S. Zhao, Eds) *Multi-disciplinary Study of the Peking Man Site at Zhoukoudian*, pp. 239–240. (Beijing: Science Press, 1985).

43 Yuan, S. X., Chen, T. M., Gao, S. J. & Hu, Y. Q. Study on uranium series dating of fossil bones from Zhoukoudian sites. *Acta Anthropologica Sinica* **10,** 189–193, (1991).

44 Pei, J. X. Thermoluminescence dating of the Peking Man site and other caves. In (R. K. Wu, M. E. Ren, X. M. Zhu, Z. G. Yang, C. K. Hu, Z. C. Kong, Y. Y. Xie & S. S. Zhao, Eds) *Multi-disciplinary Study of the Peking Man Site at Zhoukoudian*, pp. 256–260. (Beijing: Science Press, 1985).

45 Huang, P. H. *et al*. Study of ESR dating for burying age of the first skull of Peking Man and chronological scale of the cave deposit in Zhoukoudian site Loc. 1. *Acta Anthropologica Sinica* **10,** 107–115, (1991).

46 Shen, G. *et al.* High-precision U-series dating of Locality 1 at Zhoukoudian, China. *Journal of Human Evolution* **41**, 679-688 (2001).

47 Shen, G., Gao, X., Gao, B. & Granger, D. E. Age of Zhoukoudian *Homo erectus* determined with 26Al/10Be burial dating. *Nature* **458**, 198-200 (2009).

48 Zdansky, O. Preliminary notice on two teeth of a hominid from a cave in Chihli (China). *Bulletin of the Geological Society of China* **5**, 281-284 (1926).

49 Zdansky, O. A new tooth of *Sinanthropus pekinensis*. *Acta Zoologica* **33**, 189-191 (1952).

50 Bermúdez de Castro, J.M., Rosas, A., & Nicolás, M.E. Dental remains from Atapuerca-TD6 (Gran Dolina site, Burgos, Spain). *Journal of Human Evolution* **37**, 523-566 (1999).

51 Carbonell, E. *et al*. An Early Pleistocene hominin mandible from Atapuerca-TD6, Spain. *Proc Natl Acad Sci U S A* **102**, 5674-5678 (2005).

52 Tobias, P. V. & Koenigswald, G. H. R. V. A Comparison Between the Olduvai Hominines and those of Java and some Implications for Hominid Phylogeny. *Nature* **204**, 515-518 (1964).

53 Wu, R. & Wu, X. Human fossil from Xichuan, Henan. *Vertebrata Palasiatica* **20**, 1-9 (1982).

54 Gong, X. *et al.* Human fossils found from Hualong Cave, Dongzhi County, Anhui Province. *Acta Anthropologica Sinica* **33**, 427-436 (2014).
